# Sub-^1^/_10_ exciton threshold lasers using stable self-charged perovskite quantum rods

**DOI:** 10.1126/sciadv.aeb6386

**Published:** 2026-07-24

**Authors:** Jialu Li, Xue Han, Wenjie Wang, Jinhui Wang, Tingting Zhang, Yuting Wu, Guofeng Zhang, Bin Li, Changgang Yang, Wenli Guo, Mi Zhang, Ruiyun Chen, Chengbing Qin, Jianyong Hu, Zhichun Yang, Shaoding Liu, Yue Wang, Yunan Gao, Jie Ma, Liantuan Xiao, Suotang Jia

**Affiliations:** ^1^ State Key Laboratory of Quantum Optics Technologies and Devices, Institute of Laser Spectroscopy, Collaborative Innovation Center of Extreme Optics, Shanxi University, Taiyuan 030006, China.; ^2^ Key Lab of Advanced Transducers and Intelligent Control System of Ministry of Education, Taiyuan University of Technology, Taiyuan 030024, China.; ^3^ School of Integrated Circuits (School of Microelectronics), Nanjing University of Science and Technology, Nanjing 210094, China.; ^4^ Key Laboratory of Luminescence and Optical Information, Ministry of Education, School of Physical Science and Engineering, Beijing Jiaotong University, Beijing 100871, China.

## Abstract

Colloidal quantum dots (QDs) are promising optical gain materials that require a reduction in the threshold to reach their full potential. While QD charging theoretically reduces the threshold to zero, its effectiveness has been limited by strong Auger recombination and unstable charging. In this study, we theoretically determine the optimal combination of charging number and Auger recombination to minimize the lasing threshold. Experimentally, we develop stable, self-charged perovskite quantum rods (QRs) as an alternative to QDs via the state engineering and Mn (manganese) doping strategy. A two-order-of-magnitude reduction in nonradiative Auger recombination enables QRs to support a charging number of up to 6. We then achieve QR liquid lasing with a sub-^1^/_10_ exciton threshold (an average of 0.098 excitons per QR) using 5-nanosecond pulse pumping. This threshold is exceptionally low among all reported QD lasers. These achievements demonstrate the potential of specially engineered QRs as excellent gain media and pave the way for their applications.

## Teaser

Stable self-charged perovskite quantum rods afford exceptional gain, achieving sub-^1^/_10_ exciton threshold liquid lasing.

## INTRODUCTION

Colloidal semiconductor quantum dots (QDs) have demonstrated considerable promise as laser gain media, with a plethora of research efforts focused on harnessing their unique optoelectronic characteristics (*1*–*3*). These include narrow exciton emission linewidths, high quantum yields, high thermal stability, photobleaching resistance, and tunability across the entire visible spectrum (*4*, *5*). The recent realization of electrically pumped amplified spontaneous emission reaffirms the great potential of QDs for low-cost, flexible, and on-chip integrated lasers (*6*). However, their prospective applications require further achievement in preventing overheating and lowering lasing thresholds.

Recently, the successful demonstration of the high performance of liquid lasers based on rapidly heat-dissipating QD solutions provides a promising platform (*7*–*10*). In addition, the solution-based gain media allow for more convenient tuning of laser characteristics such as wavelength and spatial mode. These advantages make QD liquid lasers attractive for applications in astronomy, medicine, and spectroscopy, including but not limited to optofluidic-based on-chip environmental monitoring, medical diagnostics, and chemical weapon detection (*11*–*14*).

Early efforts to develop QD liquid lasers were unsuccessful because of the unexpectedly fast Auger recombination of QDs. The nonradiative transfer of exciton energy to a third carrier instead of photon emission, the Auger recombination, inevitably increases the critical volume fraction required for achieving the laser regime to 0.2% (not readily accessible in QD solutions) (*15*). As a result, numerous lasing demonstrations of QDs over the past two decades have been limited to densely packed QD films (*16*–*18*). Although some studies have achieved lasing in QD solutions by introducing high-quality optical resonant cavities, their lasing thresholds remain substantially higher than those of QD film lasers (*7*, *8*, *19*–*23*). To overcome these challenges and unlock the full potential of QDs as gain media, further research is needed to develop strategies for achieving lower lasing thresholds.

For threshold reduction, the suppression of nonradiative Auger recombination is imperative. The development of Auger-engineered QDs has achieved a biexciton Auger lifetime of 2 ns by suppressing the Auger process, thereby lowering the threshold in QD film lasers (*4*). However, the Auger process remains more efficient than the radiative process and requires further reduction. In addition to reducing Auger recombination, QD charging can further lower the lasing threshold by suppressing ground-state absorption and converting biexciton gain into charged exciton gain (*4*, *16*, *24*). This decreases the number of excitons required for optical gain. Nevertheless, current charging methods, such as photochemical or electrochemical approaches involving external chemical operations during charging, can permanently damage the QD gain material, severely deteriorating its lasing efficiency (*16*, *24*, *25*). Moreover, these approaches require continuous operation to prevent charge loss from spontaneous discharge, which complicates their practical implementation (*16*, *24*, *25*). In addition, QD charging can markedly increase Auger recombination by introducing additional nonradiative Auger decay channels (*26*). This limits the charging number in each QD to usually 2 in previous works (*24*, *25*), as too many charges would completely quench photoluminescence (PL) and, consequently, the lasing of QDs. This contradicts the expected positive effects on QD lasing.

In this study, we theoretically investigate the optimal combination of charging number and Auger recombination to minimize the lasing threshold. On the basis of this insight, we design and fabricate CsPbBr_3_ perovskite quantum rods (QRs) with Auger recombination reduced by two orders of magnitude. Bromine ( ${\text{Br}}_{i}^{-}$ ) interstitial states are efficiently created in the QRs, achieving a self-charging effect. This self-charging effect does not damage the QR gain material because external chemical operations are not involved during charging. To stabilize QR charging, we develop a manganese (Mn) doping strategy that preserves the ${\text{Br}}_{i}^{-}$ states by maximizing the energy barrier for ${\text{Br}}_{i}^{-}$ migration via hybridization of Mn 3d orbitals with Pb 6s-Br 4p antibonding states. This strategy enables long-term QR self-charging with a tunable charging number. Using Mn-doped QRs charged with six electrons, we achieve a record-low sub-^1^/_10_ exciton threshold (an average of 0.098 excitons per QR) in liquid lasing with 5-ns pulse pumping.

## RESULTS

### Synergistic effects of Auger and QD charging on the lasing threshold

**Fig. 1.**
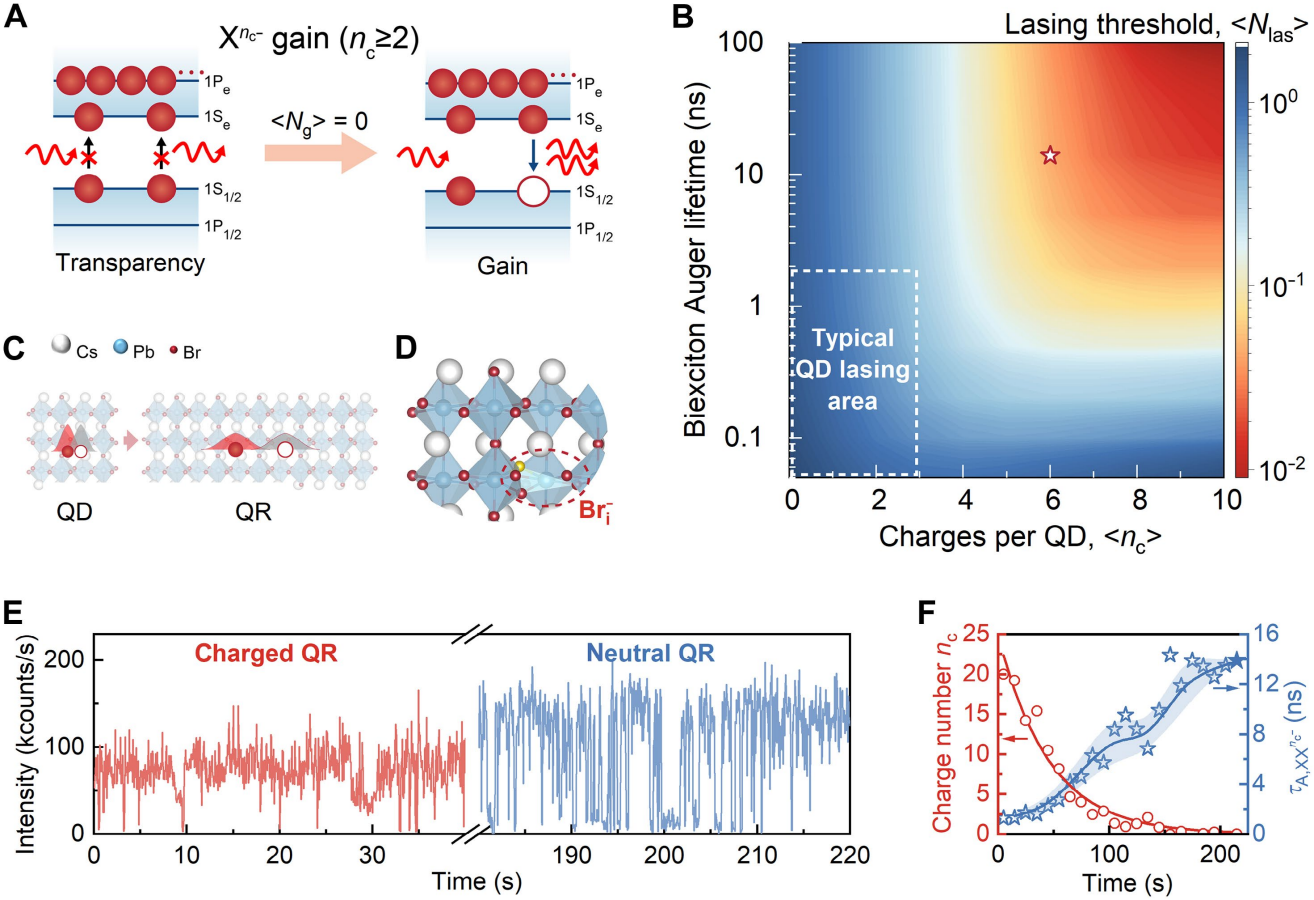
Design and characterizations of CsPbBr_3_ perovskite QRs with a self-charging effect and reduced Auger recombination. (**A**) Schematics of optical-gain mechanisms and the optical-gain thresholds $\langle N_{g}\rangle$ for multicharged exciton ( $X^{n_{c}-}$ ) gain in multicharged QDs ( $n_{c}\;\geq 2$ ). (**B**) Dependence of the lasing threshold $\langle N_{\text{las}}\rangle$ on the charging number per QD $\langle n_{c}\rangle$ and the Auger recombination (characterized by the biexciton Auger lifetime $\tau_{A,\text{XX}}$ in neutral QDs). Limited by the charging number and the Auger recombination of the typical QDs, their lasing thresholds are inside the white dashed frame. The red star represents the lasing threshold of the QR material with a charging number of 6 and an Auger lifetime of 13.9 ns in the work. (**C**) Schematics of CsPbBr_3_ perovskite QDs (left) and QRs (right). The one-dimensional elongated structure of the QR reduces the Auger recombination. (**D**) Schematic depiction of the bromine ( ${\text{Br}}_{i}^{-}$ ) interstitial state as a hole acceptor for the self-charging effect in the QR by creating a CsBr-rich environment. Excess Cs^+^, which are automatically added to the surface of the QR in its natural position ( ${\text{Cs}}_{\text{Cs}}^{+}$ ) to balance the charges of ${\text{Br}}_{i}^{-}$ , are not considered as states and are not shown for simplicity. (**E**) Typical PL intensity time trajectory for a single CsPbBr_3_ QR at excitation condition 〈N〉=0.2 . 〈N〉 represents the average exciton number per QD. The red and blue trajectories represent the time frames from 0 to 40 s for charged QR and from 180 to 220 s for neutral QR, respectively, and the whole PL trajectory from 0 to 220 s is presented in fig. S7. (**F**) Evolution of charging number (*n*_c_) (red circles) in the self-charged QR and the corresponding biexciton Auger lifetime (blue stars) over time. The decay of *n*_c_ can be well fitted by the monoexponential function. Errors are indicated by shaded areas.

Given that the band-edge states of QDs are twofold degenerate, the optical gain in QDs is achieved by biexciton gain, with an optical-gain threshold $\langle N_{g}\rangle$ of 1 in the simplified model (fig. S1A) (*24*). By charging the QD with an extra electron (*n*_c_ = 1), the ground-state absorption is partially blocked and $\langle N_{g}\rangle$ decreases sharply to 0.5 (fig. S1B) (*16*). When the charging number *n*_c_ ≥ 2, the ground-state absorption is completely blocked and $\langle N_{g}\rangle$ decreases sharply to 0 (Fig. 1A), which is achieved by the multicharged exciton ( $X^{n_{c}-}$ ) gain (*16*, *24*). However, in real situations, both the exciton number *N* and the charge number $n_{c}$ in the QD ensemble are distributed according to Poisson statistics so that $\langle N_{g}\rangle$ does not reach 0 when *n*_c_ = 2. Furthermore, entering the lasing regime requires consideration of Auger losses of the QDs and photon losses in the cavity.

Here, we theoretically calculate the dependence of the lasing threshold $\langle N_{\text{las}}\rangle$ on the Auger recombination (characterized by the biexciton Auger lifetime $\tau_{A,\text{XX}}$ ) and the charging number per QD $\langle n_{c}\rangle$ (Fig. 1B) on the basis of a modified gain-switching model that considers the Poisson distribution of $\langle n_{c}\rangle$ and the biexciton gain in charged QDs (note S1). Notably, when the QD is uncharged ( $\langle n_{c}\rangle$ = 0 in Fig. 1B), the threshold $\langle N_{\text{las}}\rangle$ changes less with increasing $\tau_{A,\text{XX}}$ . This suggests that the suppressed Auger effect alone does not effectively lower the lasing threshold in the absence of QD charging. Similarly, when $\tau_{A,\text{XX}}$ is short, increasing $\langle n_{c}\rangle$ does not markedly lower the threshold. This is because QD charging exacerbates the Auger process, counteracting the threshold-lowering effect of multicharged exciton gain. When $\tau_{A,\text{XX}}$ gradually increased, QD charging has an increasingly pronounced effect on threshold reduction. This implies that the Auger lifetime must be sufficiently long for the lowest $\langle N_{\text{las}}\rangle$ to be achieved by QD charging. Therefore, this theoretical analysis suggests that relatively high charging and effective suppression of Auger recombination in QDs must be satisfied simultaneously to achieve a lower lasing threshold.

### Design and characterization of CsPbBr_3_ QRs

On the basis of the above theoretical analysis, we design perovskite QRs to suppress the Auger process and achieve multicharge charging via a state-engineering strategy (Materials and Methods). First, to reduce the Auger recombination, we elongate the QDs to construct QRs with a higher aspect ratio (Fig. 1C). In this case, Auger recombination is reduced because exciton motion becomes diffusive, which diminishes collisions (*27*, *28*). Spatially confined electron-hole pairs transform into Coulombically bound excitons, and the scattering of carriers resulting from the Coulomb force weakens (*29*, *30*). Previous work by some of us measured the exciton binding energy of the QRs to be 34 meV (*30*), which exceeds the thermal energy at room temperature (*k*_B_*T*, where *k*_B_ is the Boltzmann constant and *T* is the temperature; ~26 meV). This means that excitons in QRs can remain stably bound at room temperature. It is worth noting that QRs still exhibit twofold-degenerated band edges as QDs (note S2), which means that QRs still retain the excellent properties of QDs as a laser gain medium. In addition, perovskite QRs are more advantageous than conventional cadmium selenide (CdSe)–based QRs because perovskite QRs are defect-tolerant, whereas CdSe QRs are susceptible to large surface trapping (*31*–*33*). Second, to achieve QR charging with sufficient electrons while avoiding QR damage, we design QRs with a self-charging effect. This is accomplished by introducing ${\text{Br}}_{i}^{-}$ interstitial states (Fig. 1D) as hole acceptors to leave extra electrons in the conduction band [fig. S5B (left)]. For this, a cesium bromide (CsBr)–rich environment is required during the QR self-assemble process, as the formation of ${\text{Br}}_{i}^{-}$ states is most energetically favorable under this condition (*34*). In addition to the single-particle spectroscopy data described below, analysis using x-ray photoelectron spectroscopy provides further experimental evidence for the presence of ${\text{Br}}_{i}^{-}$ interstitial states (see note S16 for details). Note that in conventionally synthesized QRs without a CsBr-rich environment, there is no self-charging effect (note S3).

The detailed preparation of the CsPbBr_3_ perovskite QRs is presented in Materials and Methods, and their fundamental properties are presented in fig. S6 (A to D). The average length and width of the QRs are 32 ± 4.8 and 14 ± 1.5 nm, respectively. Although their dimensions exceed the exciton Bohr radius of the bulk CsPbBr_3_ material, they still exhibit a quantum confinement effect (see note S2 for details). An average absorption cross section σ of 1.58 × 10^−13^ cm^2^ is at least one order of magnitude larger than that of previously reported QDs (*35*–*37*), which is beneficial to lasing. The PL characteristics of CsPbBr_3_ QRs are revealed by single-dot spectroscopy, which provides quantitative access to the charging number, Auger lifetimes, biexciton quantum yields, and PL stability of QRs at the single-particle level. A typical PL intensity trajectory shows an increase in PL intensity over time (Fig. 1E), contrary to the commonly reported decrease in intensity (*38*). The anomalously increased PL intensity is due to the QR transforming from charged (red region) to neutral (blue region) with increasing excitation time, which is confirmed by the radiative lifetime ratio between the two different PL regions (fig. S6, F to I, and note S6). The PL intensity of neutral QRs is higher than that of charged QRs because neutral QRs do not undergo additional nonradiative recombination processes. This anomalous increase in PL intensity is associated with the discharge of QRs and differs from the focus of other studies on ligand and defect rearrangement or photochemical reactions (see note S17 for details). We constructed a charging-associated Auger recombination model (note S7) and used it to calculate the evolution of the charging number $n_{c}$ and nonradiative Auger lifetime of the biexciton ( $\tau_{A,{\text{XX}}^{n_{c}-}}$ ) over time (Fig. 1F) on the basis of the PL data obtained above. It is revealed that the QR initially has ~20 charges (red circles), demonstrating an excellent self-charging effect. Nevertheless, under continuous illumination, the charge number (*n*_c_) gradually decreased over time, dropping from 20 charges to 0 within 200 s. As *n*_c_ decreases, $\tau_{A,{\text{XX}}^{n_{c}-}}$ prolongs because of the reduction of Auger channels (blue stars). The QR exhibits an Auger lifetime of 13.9 ns for neutral biexciton (*n*_c_ = 0, blue solid star), which is two orders of magnitude longer than that of conventional CdSe-based QDs and perovskite QDs (30 to 300 ps) (*4*, *39*). The Auger lifetime of QRs was also measured at the ensemble level using femtosecond transient absorption (TA) spectroscopy, yielding results consistent with the single-particle measurements (see note S10 for details). The longer Auger lifetime implies a effective suppression of Auger recombination, and the suppressed Auger recombination enables high PL quantum yields of charged single excitons (90% in fig. S6E) and biexcitons (98% in fig. S6F) and sufficient *n*_c_ in QRs. Both the self-charging effect and suppressed Auger process of QRs would contribute to the achievement of ultralow-threshold lasing.

The gradual decrease in charge number *n*_c_ within the QRs over time is due to the progressive loss of hole acceptors. This is because the ${\text{Br}}_{i}^{-}$ interstitial states cannot exist stably in the QRs because of the tendency for self-reorganization (*34*), where ${\text{Br}}^{-}$ naturally migrate to the QR surface and occupy their natural position ( ${\text{Br}}_{\text{Br}}^{-}$ ) to eliminate the ${\text{Br}}_{i}^{-}$ interstitial states (fig. S5E). Therefore, in this case, the self-charged QRs cannot be stably charged with multiple charges.

### Mn doping enables stable multicharge charging

**Fig. 2.**
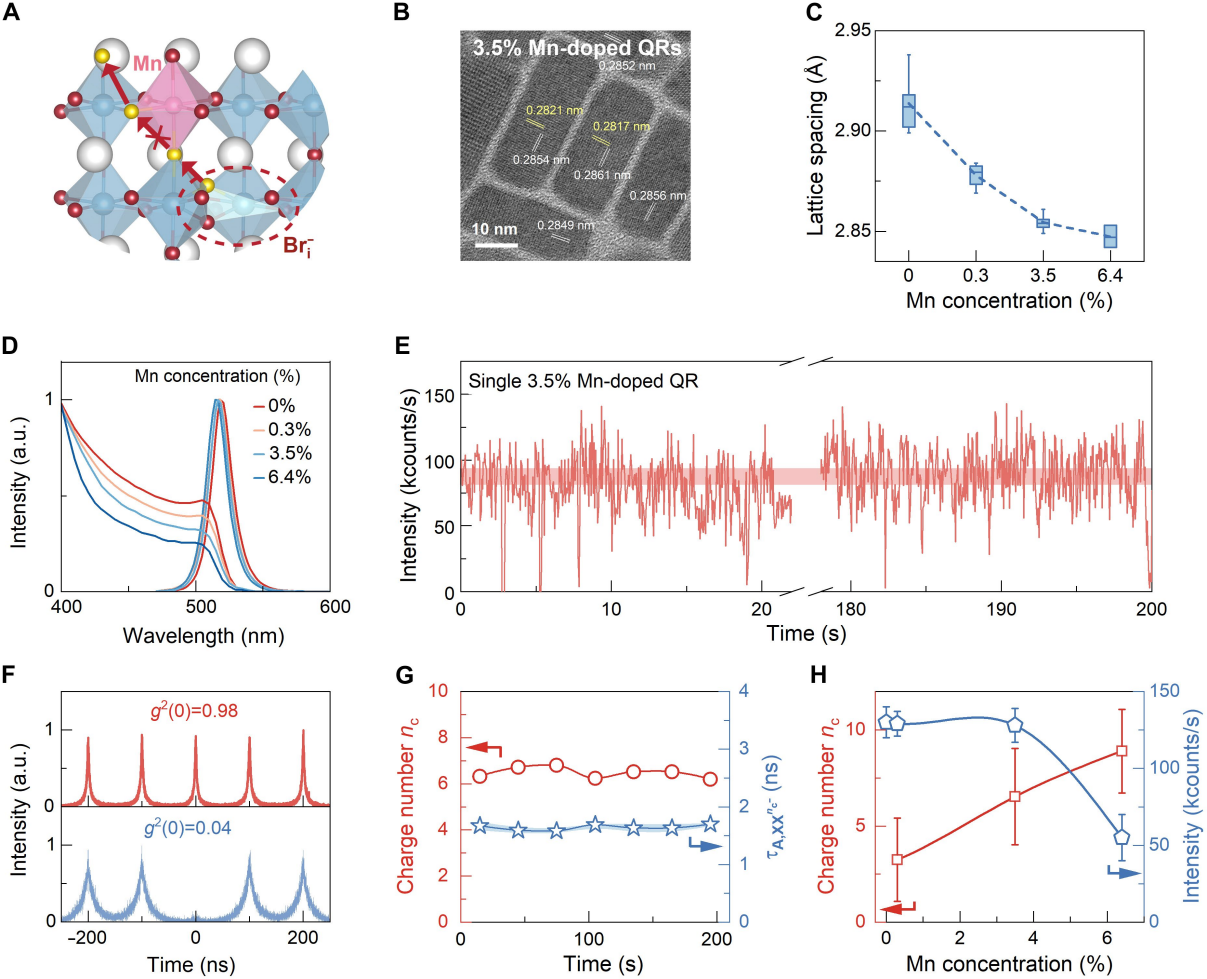
Characteristics of Mn-doped CsPbBr_3_ perovskite QRs. (**A**) Schematic depiction of ${\text{Br}}_{i}^{-}$ interstitial state migration forbidden by Mn doping–increased energy barrier, which maintains the presence of hole acceptors to stabilize QR multicharge self-charging. (**B**) High-resolution TEM (HRTEM) images of Mn-doped CsPbBr_3_ QRs with a Mn concentration of 3.5%. (**C**) Lattice spacing of CsPbBr_3_ QRs as a function of Mn doping concentration, showing lattice contraction with increasing Mn doping concentration. (**D**) Absorption and PL spectra of QRs with different Mn doping concentrations dispersed in cyclohexane. a.u., arbitrary units. (**E**) Typical PL intensity time trajectory for single 3.5% Mn-doped QRs under excitation condition 〈N〉=0.2 . (**F**) Corresponding original second-order correlation function *g*^(2)^ curve (upper panel) and time-gated *g*^(2)^ curve (lower panel) of the single QR. The *g*^(2)^(0) value of the time-gated *g*^2^ curve is 0.1, which is less than 0.5, confirming that the observed PL is derived from a single QR. (**G**) Evolutions of charging number *n*_c_ (red circles) in the QR and corresponding biexciton Auger lifetime $\tau_{A,{\text{XX}}^{n_{c}-}}$ (blue stars) over time. Shaded areas indicate errors. (**H**) Average PL intensity (blue pentagons) and charging number *n*_c_ (red squares) of ~250 single QRs with different Mn doping concentrations.

To address this issue of unstable charging, we develop a Mn doping strategy to block ${\text{Br}}^{-}$ migration along the octahedrons of the perovskite structure (Fig. 2A; Materials and Methods). The strong coordination of partially filled 3d orbitals of Mn ions with the lone-pair electrons of the Pb-Br octahedrons reduces the Pb 6s-Br 4p antibonding states and increases the energy barrier for ionic migration. The measured ionic activation energy provides direct evidence for this mechanism (see note S11 for details). Compared to other transition metal elements with empty d-orbitals, Mn has the lowest electronegativity, resulting in strong long-range lattice stabilization and optimal suppression of ionic migration (*40*, *41*). Therefore, the Mn dopant is a highly suitable candidate (see note S15 for a comparison with other elemental doping strategies). Consequently, the ${\text{Br}}_{i}^{-}$ interstitial states can stably exist as hole acceptors in Mn-doped QRs, enabling stable, multicharge, self-charging. This approach offers substantial advantages over other charging methods in terms of charging number, stability, and simplicity (see note S15 for details).

Experimentally, we dope QRs with Mn at different concentrations (0.3, 3.5, and 6.4%). The high-resolution transmission electron microscopy (TEM) images (Fig. 2B and fig. S13A) reveal the lattice contraction in Mn-doped QRs (Fig. 2C), confirming the successful incorporation of Mn into the QR lattice, which is also supported by the x-ray diffraction patterns (fig. S13B). Absorption and PL spectra of QRs with different Mn doping concentrations are shown in Fig. 2D. The gradual decrease in the first excitonic absorption peaks in the absorption spectra indicates an increase in the charging numbers with increasing Mn doping concentrations in the QRs (*42*). The PL properties of single 3.5% Mn-doped CsPbBr_3_ QRs are shown as an example (the others are shown in fig. S14). The typical PL intensity trajectory remains stable over time (Fig. 2E), in sharp contrast to that of undoped QRs (fig. S7). The *g*^(2)^(0) value of the second-order correlation function *g*^(2)^ curve is very large (upper panel of Fig. 2F). Statistical results from 70 single Mn-doped QRs indicate that their average *g*^(2)^(0) value is as high as 0.85 (fig. S15B). This is significantly larger than that of the conventional single QD (~0.1) (*43*–*46*), mainly contributed by the efficient biexciton emission resulting from the effectively suppressed Auger recombination in Mn-doped QRs (see note S6 for a detailed explanation). On the basis of the lifetimes of charged exciton ( $X^{n_{c}-}$ ) and charged biexciton ( ${\text{XX}}^{n_{c}-}$ ) obtained by fitting their decay curves (fig. S15), the charging number *n*_c_ and the Auger lifetime $\tau_{A,{\text{XX}}^{n_{c}-}}$ in the Mn-doped QRs can be calculated (see note S7 for details), as shown in Fig. 2G. It was found that approximately six stable charges were obtained in the 3.5% Mn-doped QRs. The long-term stability of these charges suggests that the Mn-doped QRs exhibit a stable, multicharge, self-charging effect. This is also verified by conductivity measurements of QR films (see note S13 for details). $\tau_{A,{\text{XX}}^{n_{c}-}}$ remains at 1.7 ns because of charging. Although $\tau_{A,{\text{XX}}^{n_{c}-}}$ decreases from 13.9 to 1.7 ns by six-charge charging, this is still comparable to the lifetimes of neutral conventional perovskite QDs (*39*, *47*) or most Auger-engineered II-to-VI QDs (*24*, *48*). When conventional QDs are charged with six electrons, their Auger lifetimes are estimated to shortened to tens of picoseconds (see fig. S12). Therefore, effectively suppressing Auger recombination is important for stable multicharge charging.

To determine the optimal Mn doping concentration, we consider the effect of Mn doping concentrations on charging numbers *n*_c_ and PL intensities. The average *n*_c_ increases with increasing Mn concentration (Fig. 2H), which is consistent with the results of absorption spectra of QRs in Fig. 2D. Moreover, we do not observe any marked change in PL intensity at Mn doping concentrations of 0.3 and 3.5% compared to the undoped QRs, while the PL intensity drops significantly at the doping concentration of 6.4% (Fig. 2H), as excess Mn doping can introduce defects to reduce the PL intensity (*49*). In addition, *n*_c_ of more than 8 in the QR has a negligible effect on the threshold reduction according to our theoretical analysis (fig. S4). As a result, the 3.5% Mn-doped QRs that exhibit high PL intensity and considerable high *n*_c_ are thus determined as the optimal gain media.

### Femtosecond TA spectroscopy and optical gain of QRs

**Fig. 3.**
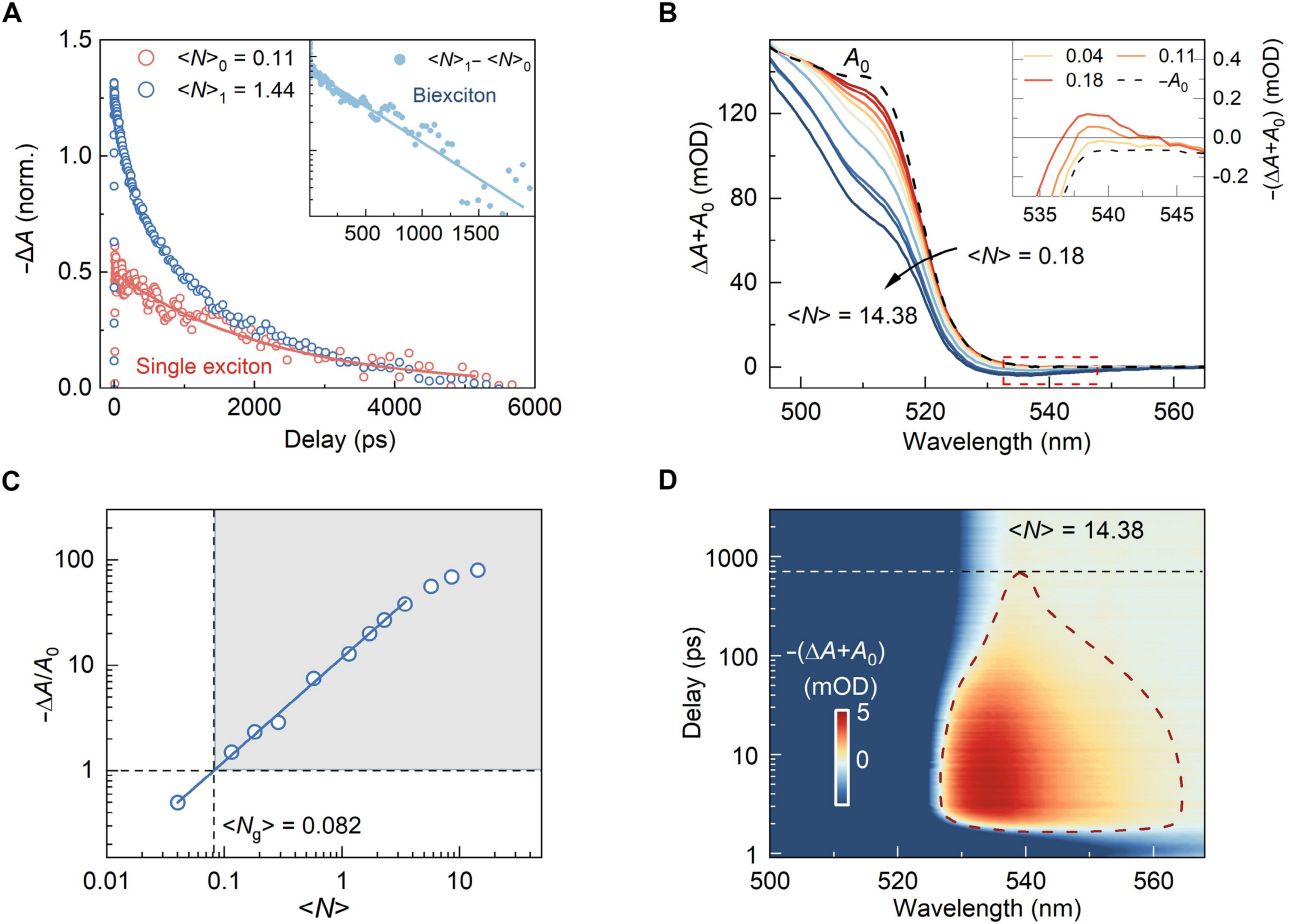
Femtosecond TA spectroscopy and optical gain of Mn-doped CsPbBr_3_ QRs. (**A**) By subtracting the exciton kinetic curve of 〈N〉 = 0.11 (red circles) from that of 〈N〉 = 1.44 (blue circles), the biexciton kinetic curve (inset) is obtained. The single exciton and biexciton kinetic curves are fitted by monoexponential functions (solid lines). (**B**) Nonlinear absorption spectra (Δ*A* + *A*_0_) at 3 ps under various 〈N〉 . Inset: Amplified absorption spectra showing the transition from absorption to net gain. (**C**) Absorption bleaching (−Δ*A*/*A*_0_) as a function of 〈N〉 for QRs indicates the gain threshold $\langle N_{g}\rangle$ = 0.082, where −Δ*A*/*A*_0_ = 1. (**D**) Two-dimensional pseudocolor plot of the net gain spectra. The gain lifetime $\tau_{g}$ is 0.735 ns at 538 nm. mOD, milli Optical Density.

To investigate the optical gain performance of QRs, we performed femtosecond TA spectroscopy measurements at different 〈N〉 (see Materials and Methods). Unlike single-dot spectroscopy, TA spectroscopy enables the study of the contributions of Auger lifetime and QR charging to optical gain at the ensemble level. Figure 3A shows the TA kinetics probed in ground-state bleach at 〈N〉 = 0.11 (red circles) and 〈N〉 = 1.44 (blue circles), normalized to their long-lived tails. The biexciton kinetic curve (inset) was obtained by subtracting the TA kinetics of 〈N〉 = 0.11 (single exciton only) from that of 〈N〉 = 1.44 (single exciton and biexciton). A monoexponential fit yields a charged biexciton lifetime of 1.2 ns, which reconfirms the reduced Auger process and the multicharge charging.

Figure 3B displays the excited-state absorption spectra (Δ*A* + *A*_0_), where Δ*A* represents the TA spectra and *A*_0_ represents the steady-state absorbance spectrum. Δ*A* + *A*_0_ decreases progressively with increasing 〈N〉 , and optical gain occurs when Δ*A* + *A*_0_ < 0 in the low-energy region (red dashed box). The inset graph clearly illustrates the transition from optical absorption to optical gain. To quantify the gain threshold $\langle N_{g}\rangle$ , we plot −Δ*A*/*A*_0_ as a function of 〈N〉 at 538 nm (Fig. 3C). The linear dependence of −Δ*A*/*A*_0_ on 〈N〉 , which deviates from the quadratic dependence of biexciton gain, strongly indicates charged exciton gain (*24*, *50*). The threshold $\langle N_{g}\rangle$ is determined to be 0.082 by extracting the horizontal coordinates of the fitted line when −Δ*A*/*A*_0_ = 1. This low threshold indicates the multicharge charging nature of QRs. Figure 3D plots the two-dimensional map of the gain and shows the time evolution of the gain spectra at 〈N〉=14.38 . The net gain region [−(Δ*A* + *A*_0_) > 0] is represented by the red dashed contour. The gain lifetime ( $\tau_{g}$ ) is determined to be 0.735 ns at 538 nm (dashed line). The $\tau_{g}$ value is comparable to those recently reported for neutral QDs with conventional structures (*7*, *10*). This benefits from substantially reduced Auger recombination and enables the use of long-duration pulses as a pump source in QR lasers.

### Sub-^1^/_10_ exciton threshold liquid lasers

**Fig. 4.**
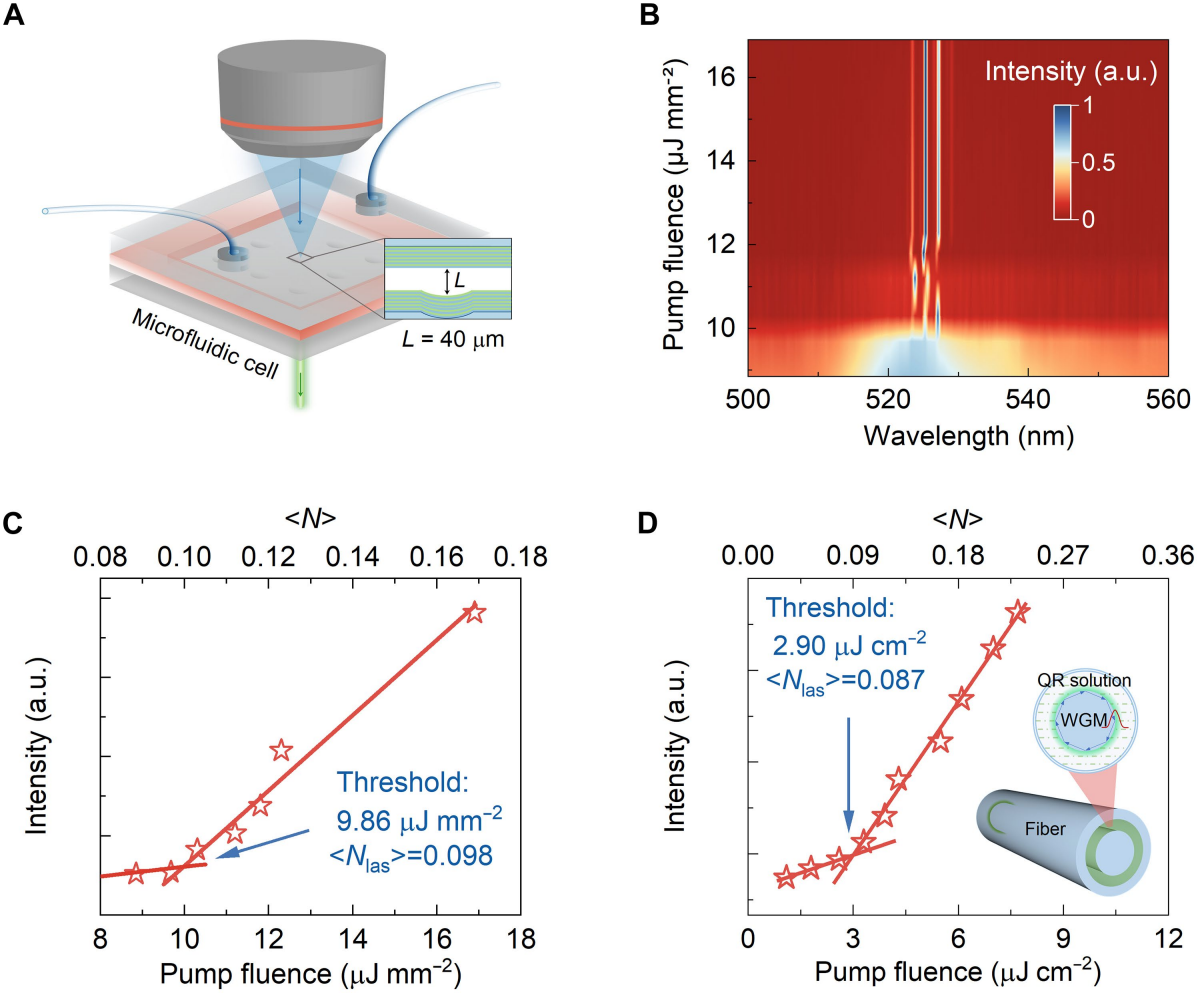
Nanosecond and femtosecond pulse-pumped QR liquid lasers. (**A**) Schematic of the F-P microcavity structure for nanosecond pulse-pumped QR lasing. It is constructed using two high-reflectivity (>99.9%) DBR mirrors, one planar and one concave, facing each other. A microfluidic channel formed between the mirrors allows for the injection of the QR solution. The cavity length *L* is ~40 μm. (**B**) Normalization of the spectral peaks versus pump fluences, which highlights the emergence of dominant laser modes with linewidths smaller than spontaneous emission. (**C**) Integrated intensity as a function of pump fluence, with the lasing threshold at 9.86 μJ/mm^2^, corresponding to 〈N〉 = 0.098. (**D**) Femtosecond pulse-pumped QR lasing: integrated intensity as a function of pump fluence, with the lasing threshold at 2.90 μJ/cm^2^, corresponding to 〈N〉 = 0.087. The inset shows the schematic of the WGM microcavity structure.

QR liquid lasing pumping with 5-ns pulses at a rate of 20 Hz is shown in Fig. 4 (A to C). The Fabry-Pérot (F-P) microcavity is constructed using two distributed Bragg reflector (DBR) mirrors (Fig. 4A). The transmission spectrum of the DBR mirrors is shown in fig. S16A, with excitation and lasing wavelengths at 478 and 525 nm, respectively. The F-P microcavity is filled with a low-concentration (0.09 μM) QR solution. Under the excitation of 5-ns pump pulses (478 nm, 20 Hz), the emission spectra exhibit a series of distinct, narrow peaks around 525.3 nm and grow rapidly with increasing pump fluences (fig. S16B). An obvious transition from spontaneous emission to lasing emission is demonstrated by the evolution of the normalized spectra with pump fluences, where the linewidth narrows significantly (Fig. 4B). A lasing linewidth at a pump fluence of 12.3 μJ/mm^2^ is determined to be 0.4 nm from a Lorentzian fitting (fig. S16C). Note that the lasing spectra in Fig. 4B exhibit red shifts compared to the PL spectra of QRs in Fig. 2D. This is attributed to the six-charged exciton gain mechanism. Charged excitons have a positive exciton binding energy, which leads to the observed red shifts in the lasing emission peaks. This finding is consistent with recent reports on charged-exciton-gain QD lasers (*16*, *24*). According to the central wavelength λ of 525.3 nm and the lasing linewidth δλ of 0.4 nm, the cavity quality factor *Q* is derived to be ~1313 by using the formula Q=λ/δλ (*19*). The rapid increase in the integrated intensity of the dominant emission peaks suggests the onset of the lasing regime (Fig. 4C). This leads to a well-defined threshold as low as 9.86 μJ/mm^2^, which is lower than those of previously reported liquid lasers (table S3) (*7*, *8*, *19*).

For the multicharged exciton gain mechanism, the lasing threshold is also expressed in terms of the excitation condition 〈N〉 (fig. S16D and note S9 for details). The threshold of 9.86 μJ/mm^2^ corresponds to an 〈N〉 value of 0.098 for the six-charged exciton gain. This means that an average of 0.098 excitons per QR is sufficient for liquid lasing onset. This is significantly lower than the 1.15 excitons required for conventional biexciton gain (*24*). Notably, the threshold of 0.098 (i.e., less than ^1^/_10_ of the exciton threshold) is lower than that reported for both QD liquid lasers (see table S3 for a detailed comparison) and QD lasers with a charged exciton gain mechanism (*4*).

The record-low threshold is further confirmed by femtosecond pulse-pumped QR lasing (Fig. 4D). Considering the inefficient optical confinement of the F-P microcavity, a capillary-tube whispering gallery mode (WGM) laser of QR solution is developed (inset). The lasing mechanism is demonstrated to be WGM by calculating the lasing modes and free spectral range (fig. S20). WGM liquid lasing occurs with increasing pump fluence (fig. S17A), where a much higher *Q*-factor of ~3495 has been achieved. The lasing threshold is obtained as 2.9 μJ/cm^2^ (Fig. 4D), which is again the lowest threshold for liquid lasers. The corresponding 〈N〉 of 0.087 at the threshold (fig. S17C and note S9 for details) reconfirms the sub-^1^/_10_ exciton threshold in QR lasing. 

## DISCUSSION

In our experiments, we achieved a sub-^1^/_10_ exciton lasing threshold in both nanosecond- and femtosecond-pumped QR liquid lasers. Our theoretical analysis in fig. S4C indicates a lasing threshold of 0.04 for six-charged exciton gain and a 13.9-ns Auger lifetime. This analysis suggests reducing the threshold further by increasing the charging number and suppressing Auger recombination. However, increasing the charging number would require developing alternative methods to stabilize QR charging because higher Mn doping concentrations introduce defects that degrade PL performance. In our work, the laser threshold is now very close to the case where the Auger lifetime is infinitely large (fig. S4C). In this case, further suppression of Auger recombination has little effect on reducing the laser threshold. Therefore, lowering the laser threshold further will require considerable effort (see note S14 for details). One possible approach would be to use a resonant pumping method that matches the energy of multiphoton excitation to specific excitonic states (*51*). This strategy would minimize quantum defects in perovskite materials. It could also reduce the required pump fluence for population inversion, facilitating the development of efficient, ultralow-threshold perovskite lasers for use in integrated photonic and bioimaging applications.

Continuous-wave and electrically pumped lasers show great potential for application. The main challenges in developing these lasers are their high pump thresholds and thermal effects. Thus far, continuous-wave lasing in colloidal QDs has primarily been achieved in densely packed QD films (*52*, *53*). However, the low volume fraction of QDs in solution makes achieving continuous-wave lasing particularly challenging. Therefore, using QR gain media in solid-film lasers should result in a lower lasing threshold. Our QR gain material exhibits a large absorption cross section of 1.58 × 10^−13^ cm^2^ (fig. S6D), a relatively long gain lifetime of 0.735 ns (Fig. 3D), and a suitable quantum confinement effect, as demonstrated by a time-gated *g*^(2)^(0) value of 0.1 (lower panel of Fig. 2F). The absorption cross section is at least one order of magnitude larger than that of previously reported QDs, making it favorable for continuous-wave lasing. The gain lifetime is comparable to that of previously reported neutral QDs, enabling the use of long-duration pump pulses in QR laser configurations. Appropriate quantum confinement ensures that QR lasers have good high-temperature stability. Together, these advantages suggest that our QR gain material has great potential for achieving continuous-wave lasing. In addition, we provide a theoretical estimation of the potential of QRs as electrically pumped laser gain materials. The threshold is 3.79 A/cm^2^ (see note S8), which is ~700 times lower than that of a conventional QD laser (2700 A/cm^2^). These results demonstrate the enormous potential of specially engineered QRs for electrically pumped lasing applications. Using the coherent Förster resonance energy transfer mechanism, Chen’s group (*54*, *55*) successfully combined energy transfer and disordered scattering on the basis of donor-acceptor QDs or hydrophobic quasi–two-dimensional perovskite microplates with tunable dimensionality, achieving electrically driven random lasers. On the basis of this strategy, electrically driven QR lasers can be fabricated by creating thin films of QRs with different bandgaps that can resonantly transfer energy between them. Our QRs, which have highly suppressed Auger losses and stable six-charged exciton gain, are promising candidates for advanced optoelectronic applications.

However, the environmental instability of perovskite materials, particularly when exposed to moisture, remains a major obstacle to their practical application in lasers. Li *et al.* (*56*) presented a promising solution to this problem: encapsulating CsPbBr_3_ perovskite QDs in a silica shell using Pb─S bonding. The composite retained strong emission in water for weeks and maintained high optical gain with low lasing thresholds. Adopting this encapsulation approach in our work could effectively enhance QR stability. This would enable the development of more robust perovskite lasers suitable for various real-world applications.

In conclusion, we have developed stable, self-charged perovskite QRs by using state engineering and Mn doping to reduce the lasing threshold through an optimal combination of Auger recombination and charging number. Using single QD spectroscopy, we demonstrate that the state engineering and Mn doping strategy enables QRs charged with six electrons. TA measurements show a low gain threshold of 0.082 and a gain lifetime of up to 0.735 ns. Using 5-ns pulse pumping, we achieved a sub-^1^/_10_ exciton lasing threshold of 0.098. Femtosecond pulse-pumped QR liquid lasing exhibited a similar threshold of 0.087. These results demonstrate the enormous potential of specially engineered QRs for lasing applications.

## MATERIALS AND METHODS

### Materials

The following materials were purchased from Aladdin: cesium carbonate (Cs_2_CO_3_; 99.0%), lead bromide (PbBr_2_; 99.0%), tetraoctylammonium bromide (98.0%), manganese bromide (MnBr_2_; 98.0%), 1-octadecene (ODE; >90%, GC), oleic acid (OA; analytical reagent), oleylamine (80 to 90%), and cyclohexane (≥99.9%). Polymethylmethacrylate and toluene (≥99.5%) were purchased from Sigma-Aldrich. Acetone was purchased from Sinopharm Chemical Reagent Co., Ltd. All materials were used as received without further purification.

### Preparation of cesium oleate solution

Cs_2_CO_3_ (0.16 g), ODE (16 ml), and OA (1 ml) were added to a 50-ml three-necked flask. Drying the mixture at 120°C for 30 min under vacuum, followed by heating to 150°C under nitrogen, forms a clear solution.

### Synthesis and purification of CsPbBr_3_ QRs

The synthesis of the CsPbBr_3_ QRs was based on a water-triggered transformation process under ambient conditions. For conventionally synthesized QRs, 2 ml of water and 2 ml of a 6 mM solution of Cs_4_PbBr_6_ nanocrystals were directly injected into a 10-ml vial. The synthesis and purification of the nanocrystals are described in the next paragraph. The mixture was left for 24 hours to complete the transformation. For CsBr-rich QRs, the goal was to allow ${\text{Br}}^{-}$ to enter the QRs and occupy interstitial positions. To achieve this, 2 ml of the Cs_4_PbBr_6_ nanocrystal solution was slowly added to 2 ml of the 4 mM CsBr solution in water. To purify the CsPbBr_3_ QRs, the QRs obtained using the two methods were centrifuged at 13,000 rpm for 5 min. The precipitates were then redissolved in 2 ml of cyclohexane to form CsPbBr_3_ QR suspensions. After another centrifugation at 8000 rpm for 5 min, high-quality CsPbBr_3_ QRs were obtained for further use.

The Cs_4_PbBr_6_ nanocrystals were prepared and purified as follows: First, 0.2 mmol of PbBr_2_, 10 ml of ODE, 1 ml of OA, and 1 ml of oleylamine were loaded into a 25-ml three-necked flask. Then, the mixture was dried at 120°C for 30 min under vacuum. The mixture was heated to 140°C under nitrogen to completely dissolve PbBr_2_. Next, 4.4 ml of cesium oleate solution (150°C) was rapidly injected into the PbBr_2_ solution. Ten seconds later, the flask was immersed in an ice water bath to terminate the reaction. After centrifugation at 8000 rpm for 5 min, the solvents and excess reactants were discarded as the supernatant. The precipitates were redissolved in 10 ml of cyclohexane to form a suspension of Cs_4_PbBr_6_ nanocrystals. After centrifugation at 3000 rpm for 5 min, a high-quality product was obtained for further use.

### Doping Mn^2+^ in CsPbBr_3_ QRs

Postsynthesis Mn^2+^ doping was processed at room temperature and under ambient conditions. The Mn precursor solution was obtained by dissolving MnBr_2_ in an acetone-toluene mixture (1:3, v/v) using sonication. With continuous stirring, the Mn^2+^ precursor solution was then added to the QR solution (1 ml) for 1 min. The amount of MnBr_2_ precursor solution added to the QR solution regulated the dopant concentration (table S2). After doping, the Mn-doped QRs were isolated by centrifugation at 13,000 rpm for 5 min. The supernatant was discarded, and the precipitate was redispersed in 1 ml of cyclohexane to obtain high-quality Mn-doped CsPbBr_3_ QRs.

### Sample preparation for single QR spectroscopic measurements

For single QR measurements, the cyclohexane QR solution was first diluted and then spin-coated onto clean glass coverslips at 3000 rpm for 1 min. Next, a polymethylmethacrylate film was spin-coated onto the single QRs to isolate and protect them.

### F-P microcavity fabrication

For lasing measurements, an F-P microcavity consisting of one planar DBR mirror and one concave DBR mirror facing each other at a distance of 30 μm was constructed. The mirrors have a maximum reflectivity of ≥99.9% at a center wavelength of 525 nm, and a copper layer separates them. The concave microstructure was fabricated using a CO_2_ (carbon dioxide) laser. A microfluidic channel was formed simultaneously, as shown in Fig. 4A. A syringe pump injects the gain medium solution, allowing it to flow through the F-P microcavity structure. For PL saturation curve measurements in liquid, the F-P microcavity mirrors were replaced with uncoated, transparent fused silica substrates. The other experimental parameters remained the same.

### Characteristics

The size and lattice spacing of the QRs were obtained from TEM images using a JEM-2100 microscope. Absorption and PL emission spectra of the QRs in cyclohexane were recorded from 300 to 800 nm using a PerkinElmer Lambda 950 ultraviolet-visible-near-infrared spectrometer and a Cary Eclipse fluorescence spectrophotometer, respectively. The mole concentration of the CsPbBr_3_ QRs and Mn dopants was determined by inductively coupled plasma mass spectrometry (ICP-MS; NexION 350X, PerkinElmer, US). Details of the ICP-MS measurements are shown in note S12. XRD patterns were measured using a D2 PHASER diffractometer.

### Single-dot PL spectroscopic measurements

We collected PL photons from single QRs using a homemade confocal fluorescence imaging microscope. We excited the QRs using a pulsed laser at 439 nm (WL-SC-400-15-PP, NKT Photonics), which had a repetition rate of 10 MHz. An oil-immersed, high–numerical aperture objective lens (Olympus, 100×, 1.3 numerical aperture) focused the laser beam onto the QR sample, collecting the PL simultaneously. The laser beam was directed into an inverted microscope (Olympus IX71), where a dichroic mirror (Semrock, Di03-R405) reflected the beam onto the sample. The mirror focused the beam on the sample through the objective. The PL, collected by the same objective, passed through the dichroic mirror and a high-pass filter (Semrock, BLP01-405R) before being focused on a 100-μm pinhole to reject out-of-focus photons. The PL was then split by a 50/50 beam splitter cube into two beams, which were detected by a pair of single-photon avalanche diode detectors (SPCM-AQR-15, PerkinElmer). A TTTR-TCSPC data acquisition card (HydraHarp 400, PicoQuant) with a temporal resolution of 16 ps recorded the PL information. The data were analyzed using a MATLAB routine. A piezo-scan stage (T-405-01, Piezosystem Jena) with an active X-Y-Z feedback loop was mounted on the inverted microscope to scan the sample over the focused excitation spot. All measurements were performed at room temperature.

### TA measurements

TA measurements are performed on QR solutions in an airtight cuvette. A femtosecond fiber laser (YF-FL-10-IR/GN/UV, Hangzhou Yacto Technology) generates 290-fs photon pulses at a wavelength of 1030 nm and a frequency of 20 kHz. The laser output is divided into two parts. The 343-nm pump pulse is generated from the main portion using triple-frequency methods. The remaining portion is focused on an yttrium-aluminum-garnet crystal to generate a supercontinuum probe beam. An off-axis parabolic mirror focuses the white light onto the sample. The probe beam that passes through the sample is collected by a series of lenses and focused onto a spectrometer. A motorized stage controls the time delay between the pump and probe beams. The pump pulses are modulated by an asynchronous chopper, and transient signals are obtained by calculating the absorption changes of two adjacent probe pulses. All experimental data are corrected for the chirp induced by the nonlinear white light generation process. All experiments are performed at a room temperature of 20°C.

### Lasing measurements

The QR liquid lasers were pumped for lasing measurements using either a nanosecond pulsed laser with a wavelength of 478 nm, a pulse width of 5 ns, and a repetition rate of 20 Hz or a femtosecond pulsed laser with a wavelength of 400 nm, a pulse width of 100 fs, and a repetition rate of 1 kHz. The diameter of the focused nanosecond pulsed laser spot is ~360 μm. The diameter of the focused femtosecond pulsed laser spot is ~70 μm. The absorption cross section of the QR ensemble in solution is ~1.94 × 10^−14^ cm^2^ (see note S9 for details). The pump energy could be adjusted using a neutral density attenuator. The molar concentration of the QR solution is 0.9 μM, as determined by ICP-MS. The QR emission signal in the cavity was collected with an optical lens and sent to a spectrometer (Horiba 320 for nanosecond pulse-pumped QR lasing with a resolution of 0.128 nm; Andor SR750 for femtosecond pulse-pumped QR lasing with a resolution of 0.0555 nm). All lasing experiments were conducted at a room temperature of 20°C.

## Supplementary Materials

### This PDF file includes:

Figs. S1 to S22 Notes S1 to S17 Tables S1 to S3 References
