# Supplementary material for: Sub-1/10 exciton threshold lasers using stable self-charged perovskite quantum rods
Source: Sci Adv. 2026 Jul 24;12(30):eaeb6386. doi: 10.1126/sciadv.aeb6386 (PMC13398479; doi:10.1126/sciadv.aeb6386)
Supplement: Supplementary file 1 — Figs. S1 to S22 Notes S1 to S17 Tables S1 to S3 References [file sciadv.aeb6386_sm.pdf]

Science Advances
AAAS

Supplementary Materials for

Sub-$^{1}/_{10}$ exciton threshold lasers using stable self-charged perovskite quantum rods

Jialu Li *et al.*

Corresponding author: Guofeng Zhang, guofeng.zhang@sxu.edu.cn; Shaoding Liu, liushaoding@tyut.edu.cn; Yue Wang, ywang@njust.edu.cn; Yunan Gao, yngao@bjtu.edu.cn; Liantuan Xiao, xlt@sxu.edu.cn

Sci. Adv. **12**, eaeb6386 (2026)
DOI: 10.1126/sciadv.aeb6386

This PDF file includes:

Figs. S1 to S22
Notes S1 to S17
Tables S1 to S3
References

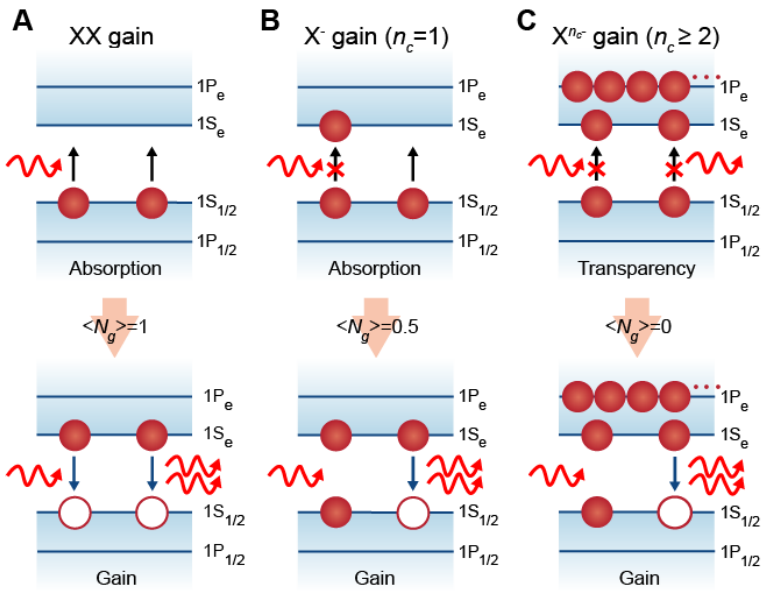

Fig. S1. Schematics of optical-gain mechanisms. The optical-gain thresholds $\langle N_g \rangle$ for (A) biexciton gain in neutral quantum dots (QDs), (B) single-charged exciton (X^-) gain in single-charged QDs and (C) multi-charged exciton (X^{n_c-}) gain in multi-charged QDs ($n_c \geq 2$).

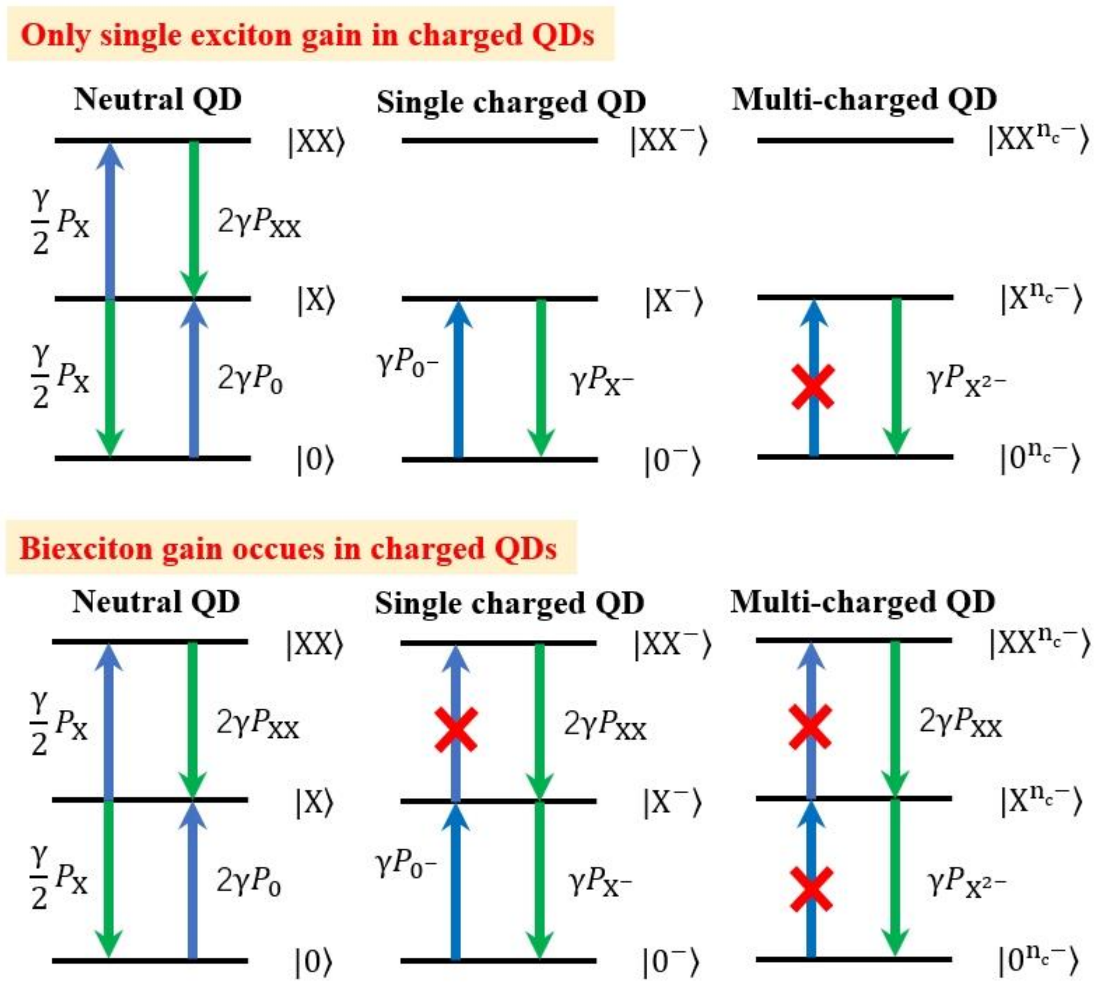

Fig. S2. Schematics of electronic states and optical transitions considering charged exciton gain and biexciton gain in charged QDs. Optical transitions (blue arrows indicate absorption and green arrows indicate stimulated emission) are shown for neutral QDs, single charged QDs, and multi-charged QDs, and their transition rates are labelled next to the respective transitions.

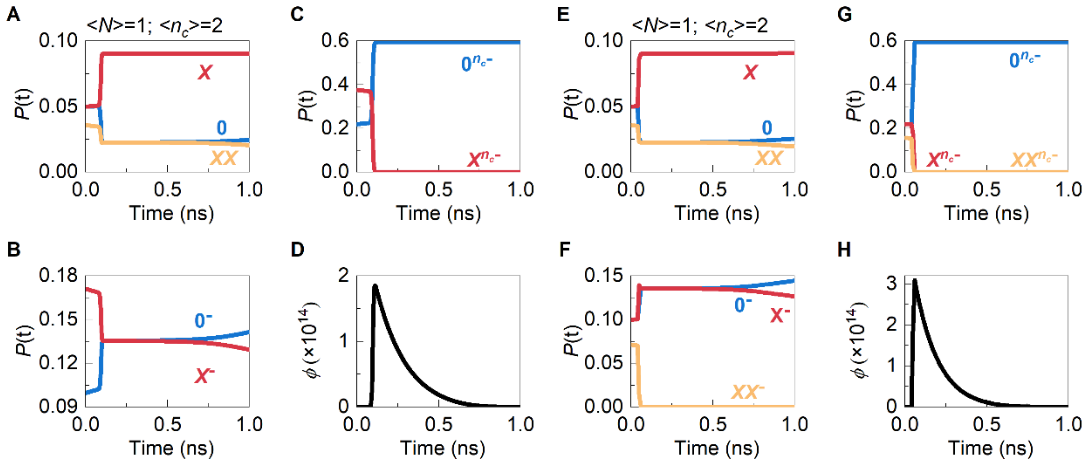

Fig. S3. Temporal evolutions of fractions of the biexciton state (P_{XX}), exciton state (P_X) and ground state (P_0), as well as the photon density in the cavity (ϕ) at excitation condition $\langle N \rangle$ = 1 and charging charges per QD n_c = 2. (A-D) Considering only charged exciton gain in the charged QRs, corresponding P_{XX}, P_X and P_0 for (A) neutral QRs, (B) single charged QRs, (C) multi-charged QRs, and (D) ϕ. (E-H) Considering both charged exciton gain and biexciton gain in charged QRs, corresponding P_{XX}, P_X, and P_0 for (E) neutral QRs, (F) single charged QRs, (G) multi-charged QRs, and (H) ϕ.

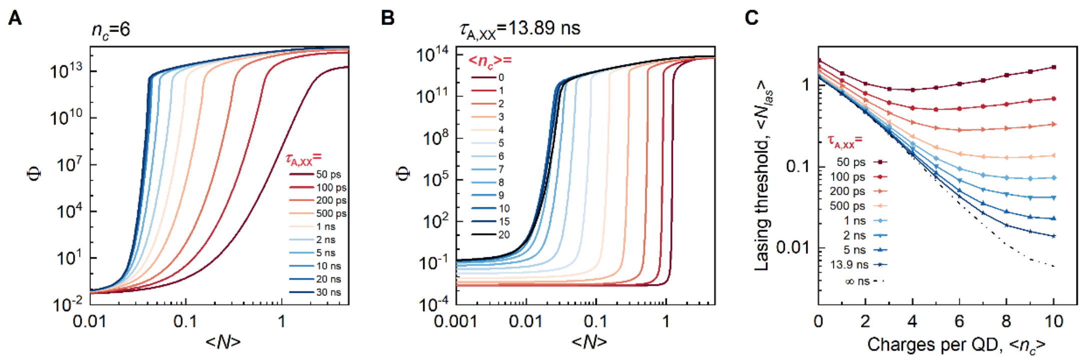

Fig. S4. Time integrated photon density and lasing threshold depend on the excitation conditions, the charging numbers, and the biexciton Auger lifetimes. (A) Time integrated photon density Φ as a function of $\langle N \rangle$ at a constant charge number of 6 and various biexciton Auger lifetimes ($\tau_{\text{A,XX}}$). (B) Φ as a function of $\langle N \rangle$ at various charge numbers and a constant biexciton Auger lifetime of 13.89 ns. When the charging charges possessed by QRs reach 6 and the biexciton Auger lifetime exceeds 10 ns, further increasing the lifetime has little effect on the threshold. Additionally, when the QRs' biexciton Auger lifetime is constant at 13.89 ns, further increases in charge number greater than 8 have little effect on the threshold. (C) Dependence of the lasing threshold $\langle N_{las} \rangle$ on the charging number per QD $\langle n_c \rangle$ and the Auger recombination (characterized by $\tau_{\text{A,XX}}$ in neutral QDs).

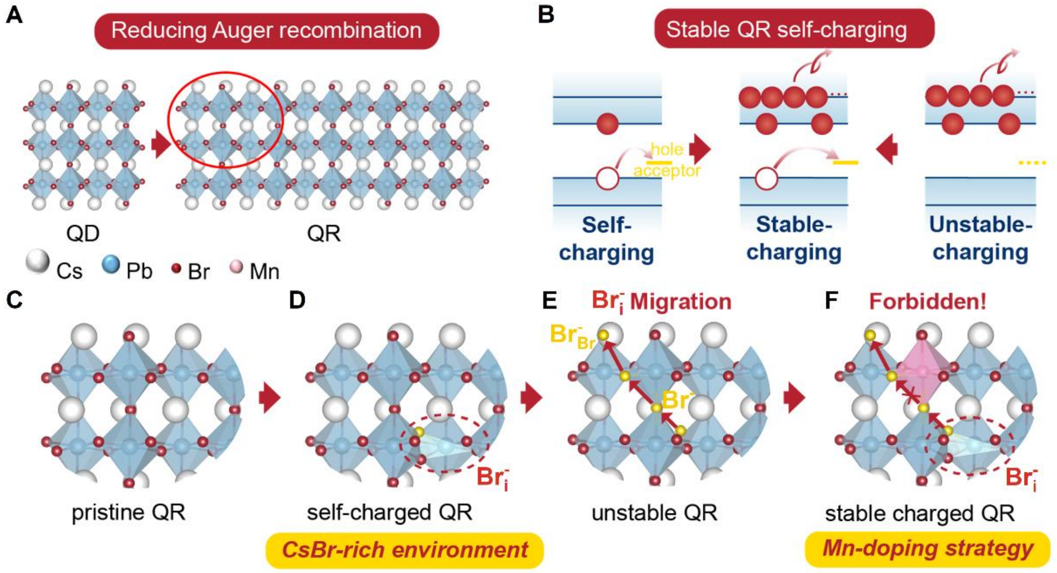

Fig. S5. Schematic illustration of Auger suppression and the stable self-charging effect. (A) Top view of $CsPbBr_3$ perovskite QDs (left) and quantum rods (QRs) (right). One-dimensional elongated QRs can reduce Auger recombination. (B) Schematic of the design idea for stable self-charged QRs by state engineering. The states are constructed as hole acceptors to generate self-charging effect (left). Unstable hole acceptors lead to failure of the self-charging effect (right). Maintaining the presence of hole acceptors ensures the stable self-charging (middle). Spiral arrows indicate spontaneous discharging processes. (C) Enlarged view of pristine QRs in the red circle region of fig. S5A. (D) Schematic depiction of bromine (Br_i^-) interstitial states as hole acceptors in the self-charged QRs by establishing a CsBr-rich environment. Excess Cs^+, which automatically add to the QR surface in its natural position (Cs_{Cs}^+) to compensate the charges of Br_i^- , are not considered as states and not shown for simplification reason. (E) Schematic depiction of the migration of Br^- to its

natural position on the QR surface, as the QR tries to reorganize itself, ultimately leading to the elimination of the hole acceptors. (F) Schematic depiction of Br^- migration forbidden by Mn-doping-increased energy barrier, which maintains the presence of hole acceptors to stabilize QR charging.

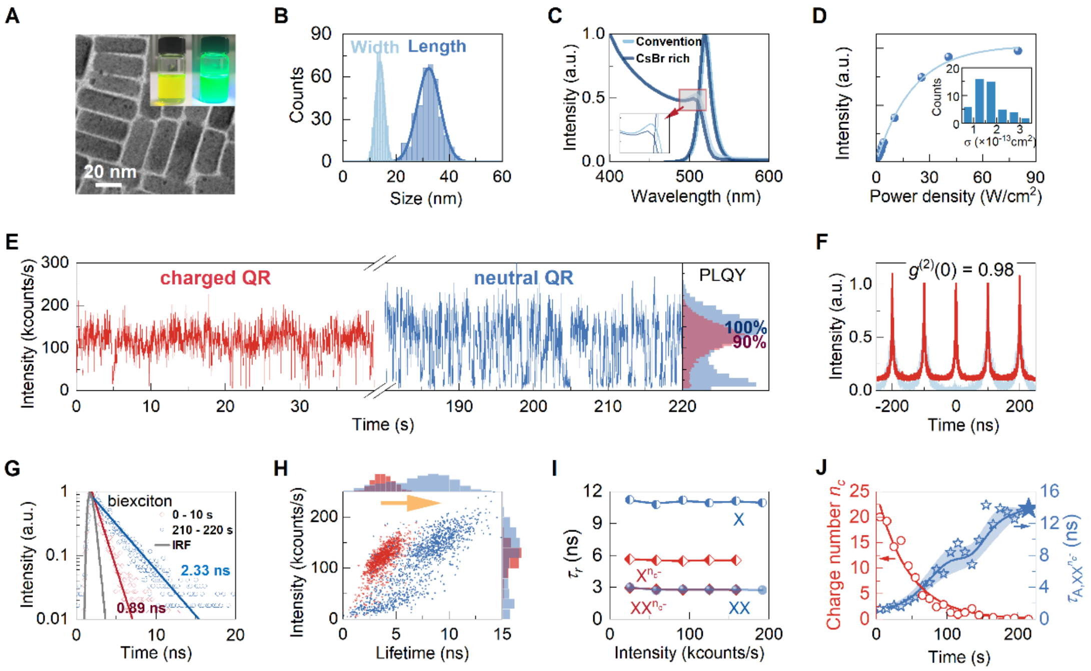

Fig. S6. Characteristics of undoped $CsPbBr_3$ perovskite QRs. (A) Transmission electron microscopy (TEM) images of undoped $CsPbBr_3$ perovskite QRs. Insets: photographs of the QR solution under room and UV light. (B) Size histograms of $CsPbBr_3$ QRs with lengths of 32 ± 4.8 nm and widths of 14 ± 1.5 nm. (C) Absorption and photoluminescence (PL) spectra of QRs dispersed in cyclohexane. The PL spectra of the conventional and CsBr-rich QRs are centered at 521 and 519 nm, respectively, with a narrow full width at half maximum (FWHM) of 15 nm. (D) A typical PL saturation curve of a single QR. Inset: Histogram of the absorption cross sections (σ) of single QRs showing a mean value of 1.58 × 10^{-13} cm^2. (E) A typical PL intensity time trajectory for a single undoped $CsPbBr_3$ QR at an excitation condition $\langle N \rangle$ = 0.2. The red and blue regions represent the time frames from 0 to 40 s and from 180 to 220 s of the PL trajectory, respectively. The entire PL trajectory from 0 to 220 s is presented in fig. S7. The corresponding PL intensity histograms of the two PL regions are shown in

the right panel. (F) Corresponding original second-order correlation function, $g^{(2)}$ (red), and time-gated $g^{(2)}$ (blue) curves. (G) Corresponding PL decay curves of the biexcitons extracted from the bright states of the PL regions marked in respective colors and fitted by mono-exponential functions. IRF represents the instrument response function. (H) Corresponding fluorescence lifetime-intensity distribution (FLID) maps for the two PL regions. The PL intensity and lifetime histograms are in the top and right panels, respectively. The lifetime values, mainly contributed by single excitons, are obtained by mono-exponential fitting of each 30 ms time bin of the PL trajectory. (I) Corresponding radiative lifetime values (τ_r) of the biexciton and single exciton as a function of PL intensity for the two PL regions. (J) Evolution of the charging number n_c (red circles) in the self-charged QR and the corresponding biexciton Auger lifetime (blue stars) over time. Errors are indicated by shaded areas. The constant decay of n_c is caused by the loss of hole acceptors.

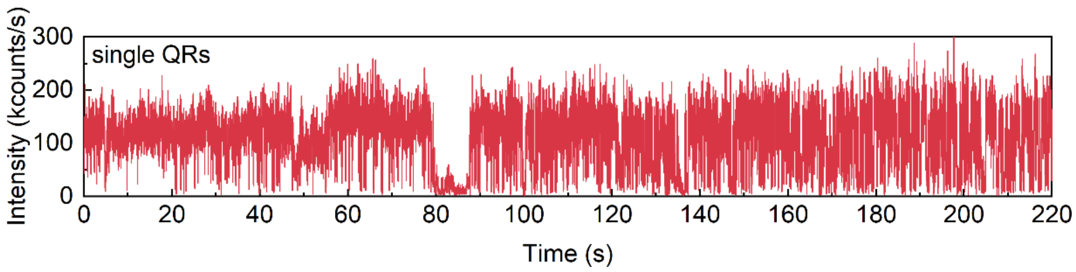

Fig. S7. The entire PL intensity trajectory of a typical single undoped $CsPbBr_3$ QR (only the first 40 s and the last 40 s are shown in Fig. 1E).

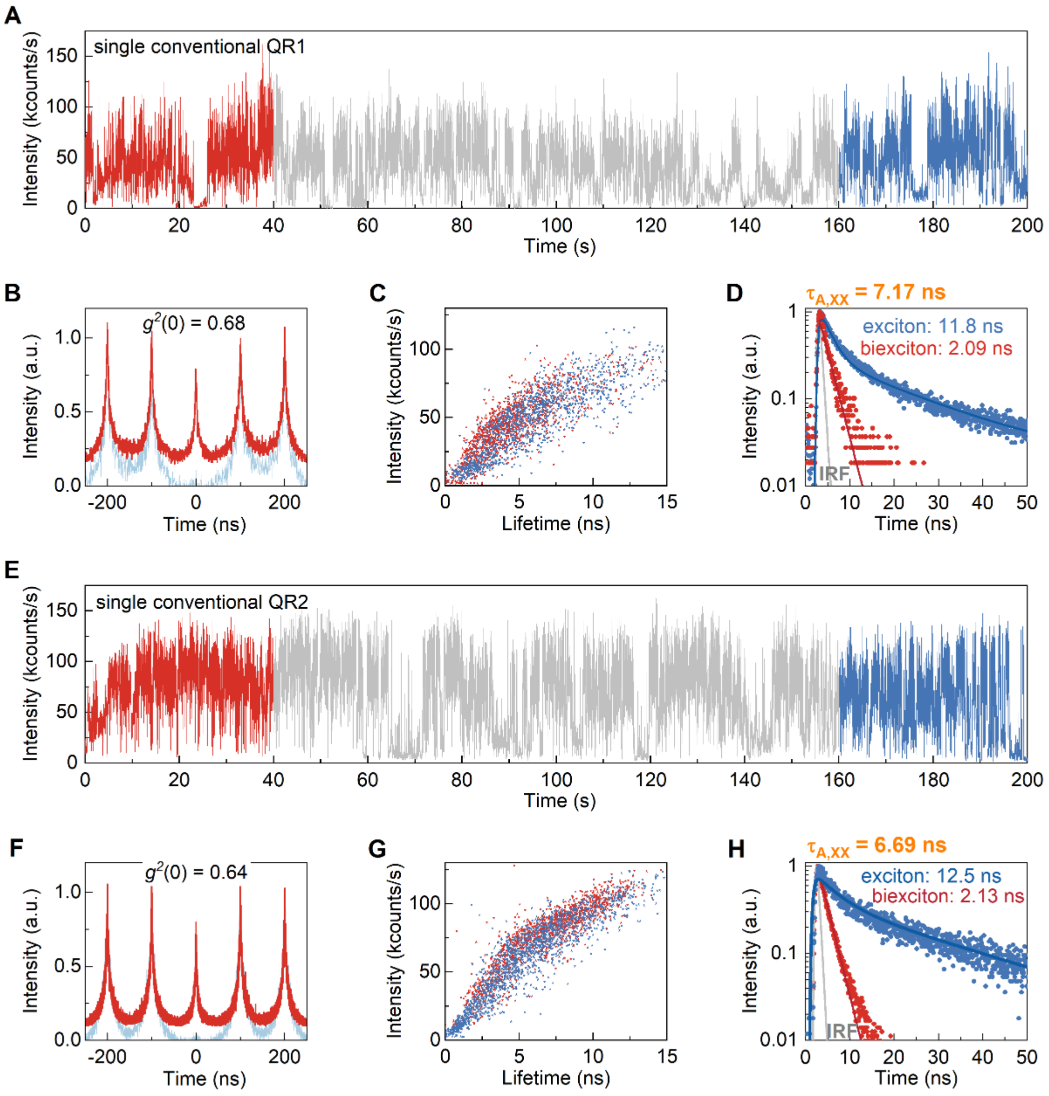

Fig. S8. Characteristics of conventional $CsPbBr_3$ perovskite QRs. (A, E) Typical PL intensity time trajectories for single conventional QRs. The red and blue regions represent the first 40 s and the last 40 s of the PL trajectories, respectively. (B, F) Corresponding original $g^{(2)}$ (red) and time-gated $g^{(2)}$ (blue) curves. (C, G) Corresponding FLID maps for the red and blue PL regions. (D, H) Corresponding PL decay curves of the single exciton (blue) and the biexciton (red), which were extracted from the bright state of their PL trajectories.

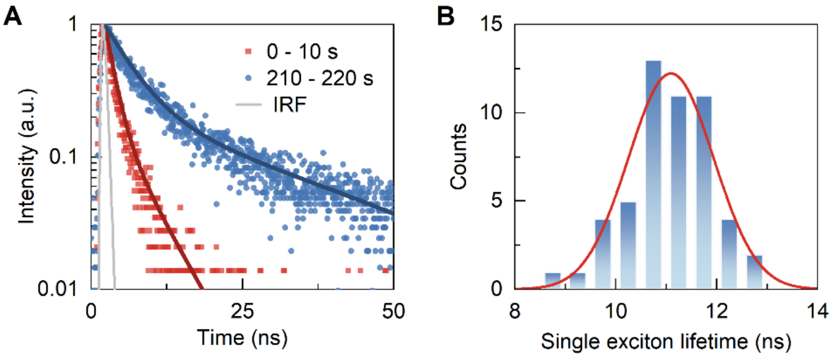

Fig. S9. Typical PL decay curves of typical single undoped $CsPbBr_3$ QR in fig. S6 and histogram of single exciton lifetime. (A) Corresponding PL decay curves obtained from the PL regions marked in fig. S6E, shown in their respective colors. These curves were fitted using a biexponential function, where the short components represent the biexciton lifetime and the long components represent the single exciton lifetime. Significant prolongations of the lifetime are found in both the single exciton and the biexciton. (B) Histogram of neutral single exciton lifetimes for 52 QRs obtained from the long components of the blue PL decay curves.

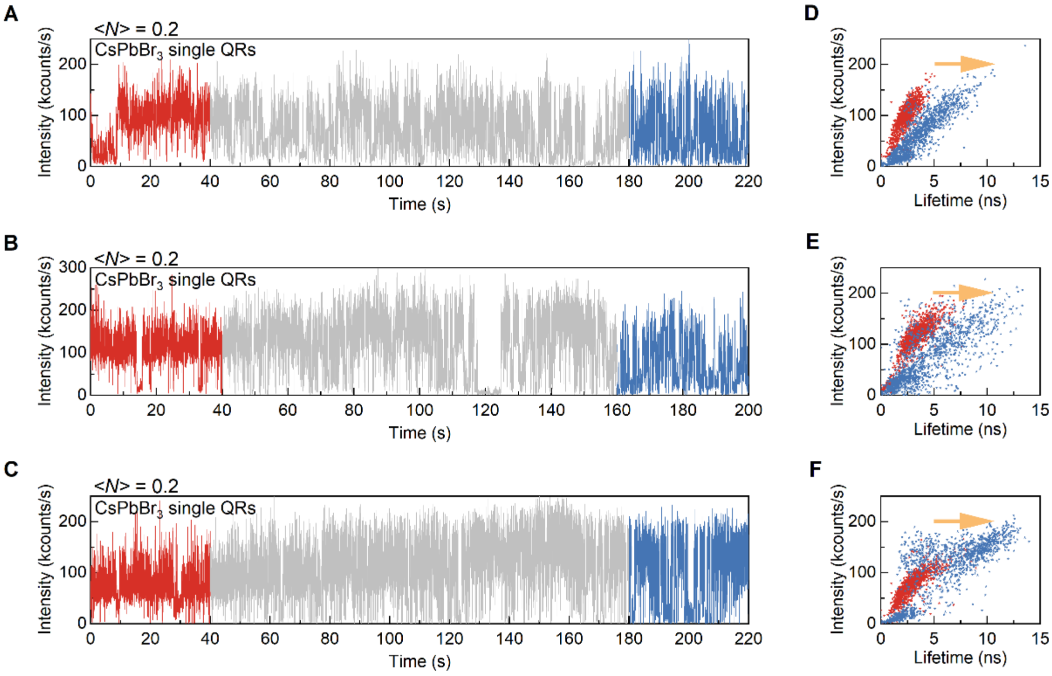

Fig. S10. Typical single-dot measurement results of single undoped $CsPbBr_3$ QRs. (A-C) Typical PL intensity time trajectories for single undoped $CsPbBr_3$ QRs at $\langle N \rangle$ = 0.2. The red and blue regions represent the first 40 s and the last 40 s of the PL trajectories, respectively. (D-F) Corresponding FLID maps for the red and blue PL regions, indicating a change in the lifetime values.

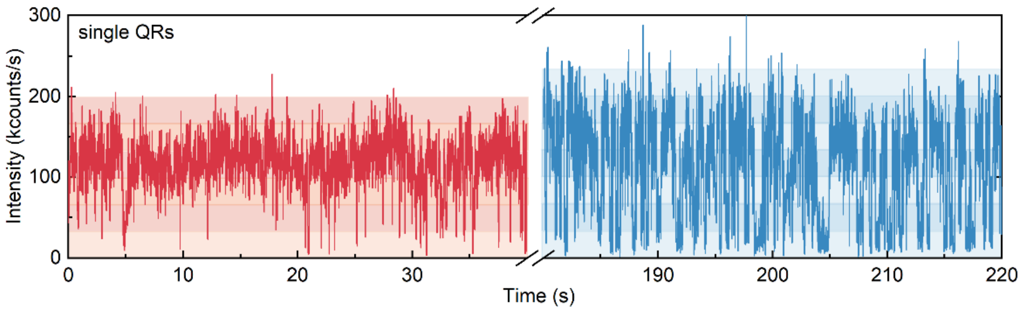

Fig. S11. Schematics of the method for extracting the second-order correlation function and decay curves of single exciton and biexciton of different PL intensity levels from the PL trajectory with an interval of 30 kcounts/s, for the determination of radiative lifetimes of single exciton and biexciton at different PL intensities.

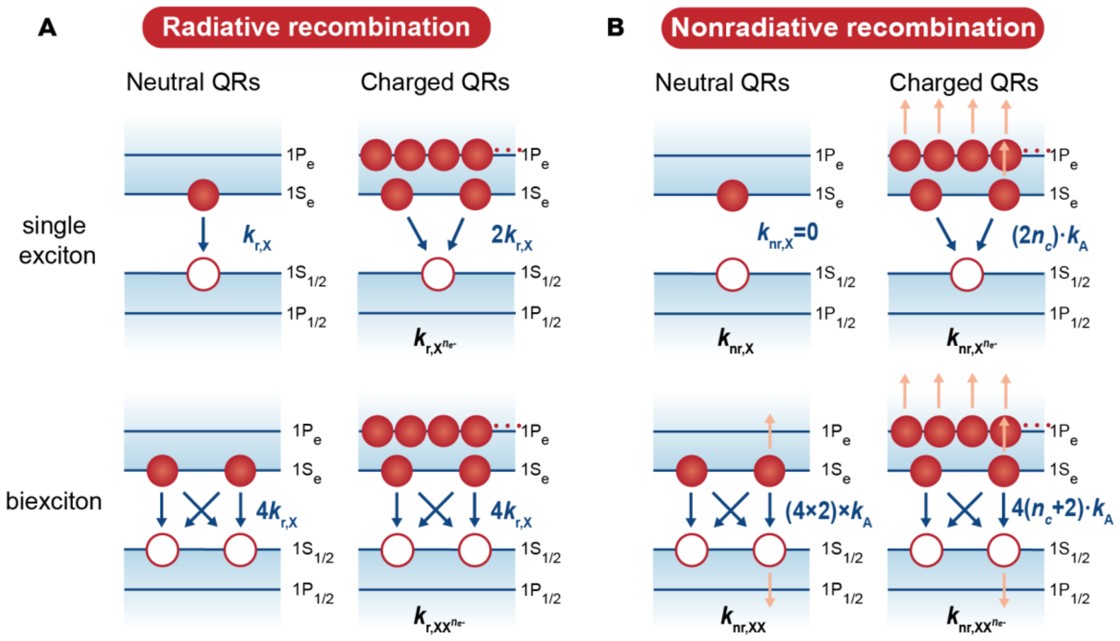

Fig. S12. Schematic of (A) radiative and (B) nonradiative recombination pathways of different excitons in the twofold-degenerate asymmetric band structure model. $k_{r,\mathrm{X}}$ represents the radiative recombination rate of single exciton X in neutral QRs. k_{A} represents the Auger rate per nonradiative recombination pathways. n_c denotes the charging number of charged QRs. Blue and orange arrows represent exciton recombination and energy transfer pathways, respectively.

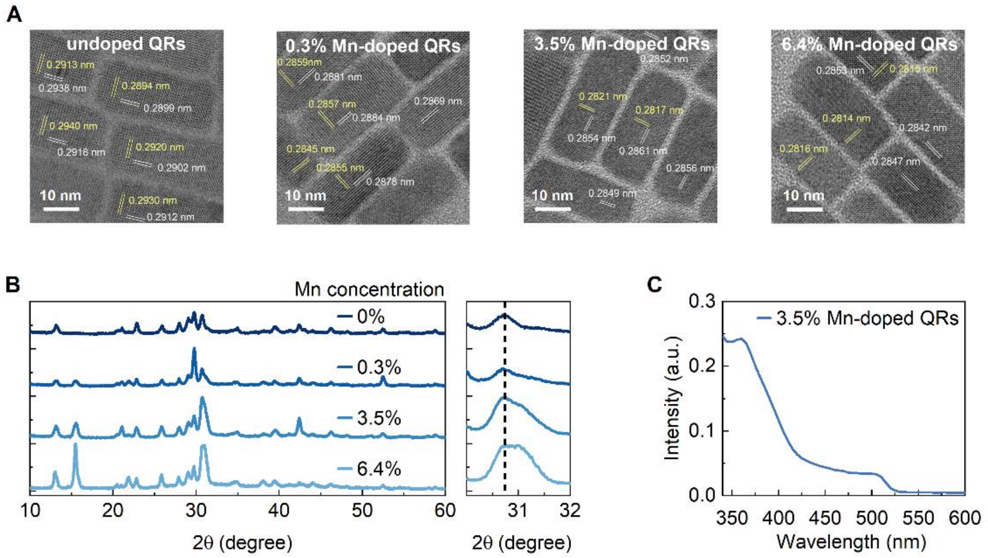

Fig. S13. Characteristics of Mn-doping $CsPbBr_3$ QRs. (A) High-resolution TEM images and (B) X-ray diffraction (XRD) patterns of $CsPbBr_3$ QRs with Mn-doping concentrations of 0%, 0.3%, 3.5% and 6.4%, respectively. (C) Absorption spectrum of 3.5% Mn-doped QRs.

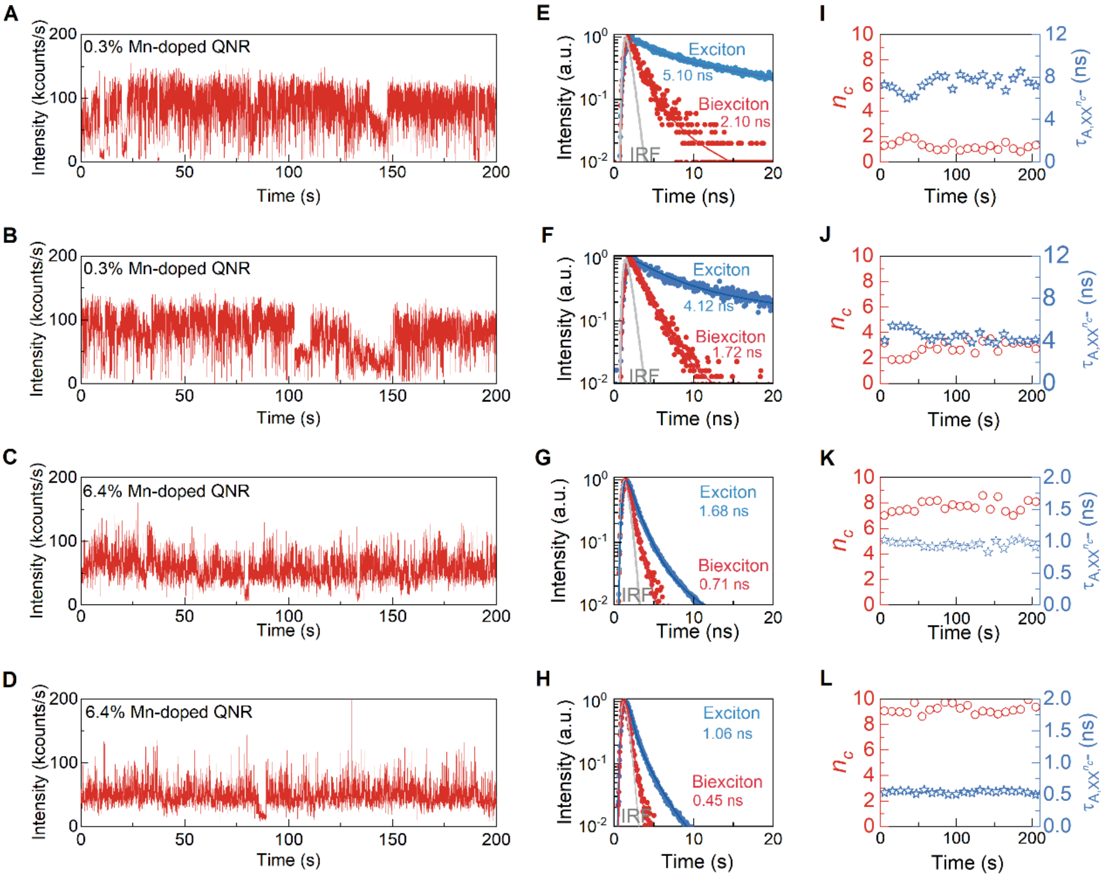

Fig. S14. PL properties of Mn-doped single QRs with Mn-doping concentrations of 0.3% and 6.4%, excited at $\langle N \rangle$ = 0.2. (A-D) Typical PL intensity time trajectories for single Mn-doped QRs. (E-H) Corresponding PL decay curves of single exciton (blue) and biexciton (red) obtained from the bright state of the PL trajectories. (I-L) Charge number n_c evolution (stars) and biexciton Auger lifetime $\tau_{\mathrm{A,XX}^{n_c-}}$ evolution (dots) over time.

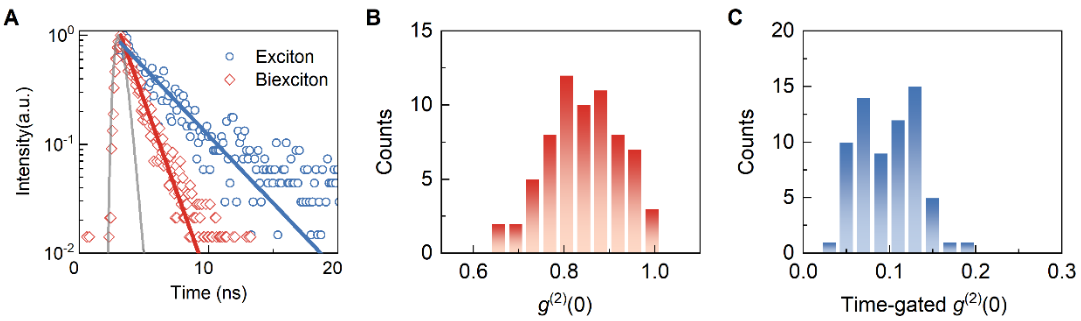

Fig. S15. Typical PL decay curves of charged exciton and biexciton, and histograms of $g^{(2)}(0)$ values of the second-order correlation function (g^2) curves. (A) Corresponding PL decay curves of charged exciton (blue) and charged biexciton (red) extracted from the bright state of the PL trajectory shown in Fig. 2E. Fitting these curves with a single exponential function yielded lifetimes of 3.36 ns for the charged exciton (X^{n_c-}) and 1.31 ns for the charged biexciton (XX^{n_c-}). (B) Histogram of the $g^{(2)}(0)$ values for 70 single Mn-doped QRs. The average $g^{(2)}(0)$ value is 0.85. (C) Histogram of the time-gated $g^{(2)}(0)$ for 70 single Mn-doped QRs. The average time-gated $g^{(2)}(0)$ value is 0.10.

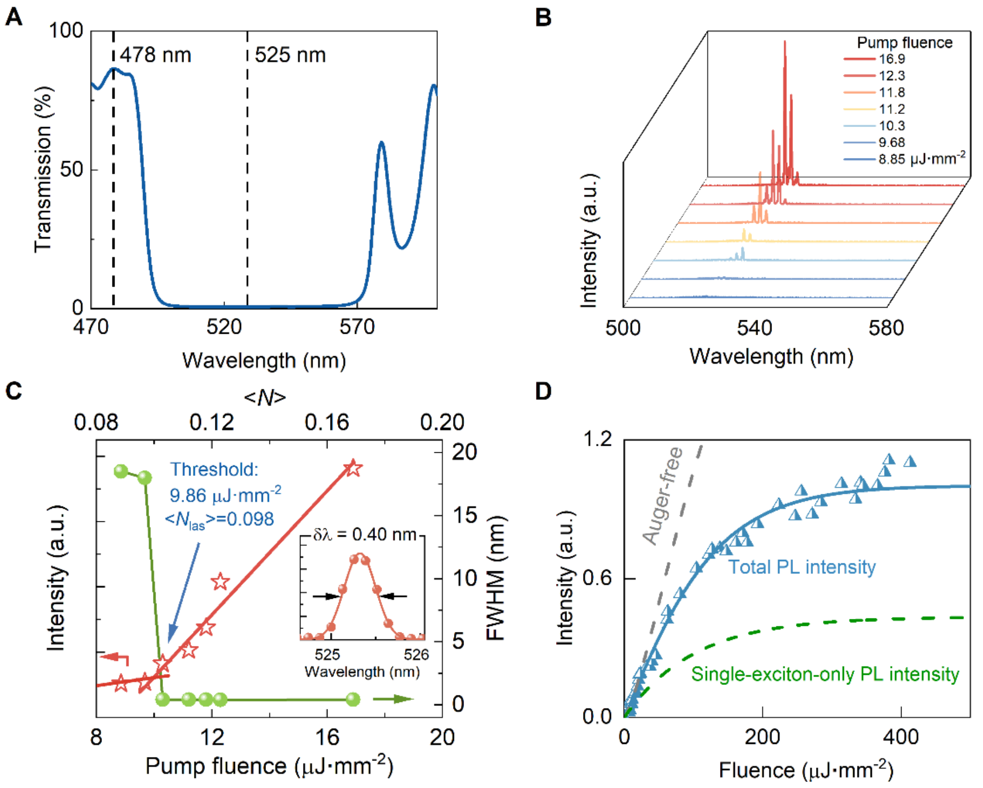

Fig. S16. Characteristics of QR liquid lasing pumping with 5-nanosecond pulses. (A) Transmission spectrum of the DBR mirrors. The spectrum shows a stopband width of ~60 nm and peak reflectivity of ~99.9%, centered at ~525 nm. The excitation (478 nm) and lasing (525 nm) wavelengths are shown with dashed lines. (B) Pump fluence-dependent emission spectra from the QR laser, which was excited with 5 ns pump pulses at 478 nm and 20 Hz. (C) Integrated emission intensity and full width at half maximum (FWHM) of the dominant emission peaks as a function of pump fluence, showing a lasing threshold of 9.86 μJ·mm^{-2}, corresponding to an average exciton number $\langle N_{las} \rangle$ of 0.098. When the pump fluence is below the threshold, the line width remains broad (~18 nm). Once the pump fluence reaches the threshold, the line width suddenly narrows to ~0.4 nm, coinciding with the superlinear growth of emission intensity (red stars). Inset: Lorentzian fitting of the lasing mode at 525.3 nm

indicates that the FWHM is approximately 0.4 nm. (D) A typical PL saturation curve of QRs (blue triangles) measured in a modified F-P microcavity structure, wherein the DBR mirrors were replaced with blank glass substrates. The correspondence between pump fluence and pump condition $\langle N \rangle$ can be obtained by fitting the PL saturation curve with a multiexciton-correlated PL saturation model (solid curve). The green and gray dashed curves are theoretical PL saturation curves with multiexciton quantum yields (QYs) of 0% (single-exciton only) and 100% (Auger-free), respectively.

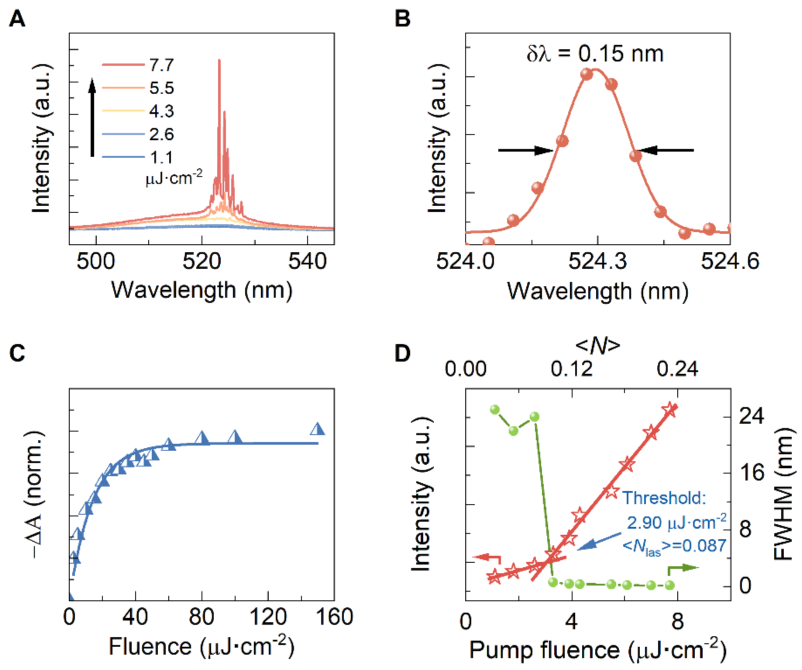

Fig. S17. Characteristics of QR liquid lasing pumping with femtosecond pulses. (A) Pump fluence-dependent emission spectra from the QR laser excited with femtosecond pump pulses at 400 nm and 1 kHz. (B) Lorentz fitting of the lasing mode at 524.3 nm indicates a FWHM of approximately 0.15 nm. (C) Pump fluence-dependent ground-state absorption at a delay time of 2 ns (only single exciton remains). The correspondence between pump fluence and $\langle N \rangle$ can be obtained by fitting the curve according to the Poisson distribution (solid curve). (D) Integrated emission intensity and full width at half maximum (FWHM) of the dominant emission peaks as a function of pump fluence. When the pump fluence is below the threshold, the line width remains broad (~24 nm), which is characteristic of spontaneous emission. Once the pump fluence reaches the threshold, the line width suddenly narrows to ~0.15 nm, coinciding with the superlinear growth of emission intensity (red stars). The abrupt narrowing of the line width and the sudden change in intensity confirm

that the lasing threshold of this system is 2.90 $\mu J \cdot cm^{-2}$, corresponding to an average exciton number $\langle N_{las} \rangle$ of 0.087.

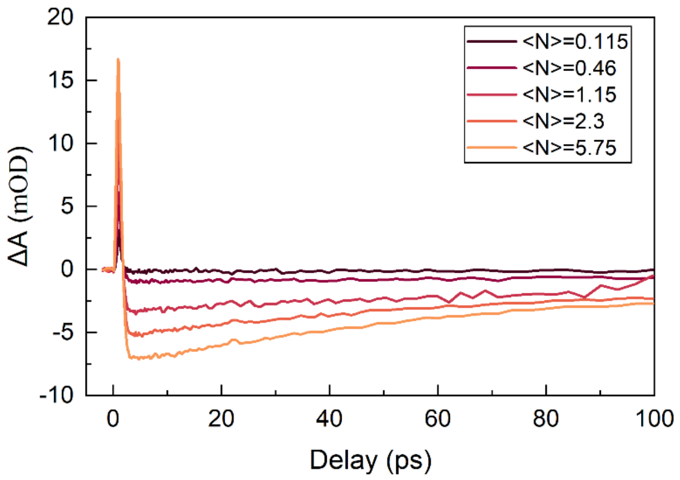

Fig. S18. Pump fluence-dependent photoinduced absorption (PA) signals of QRs under different excitations.

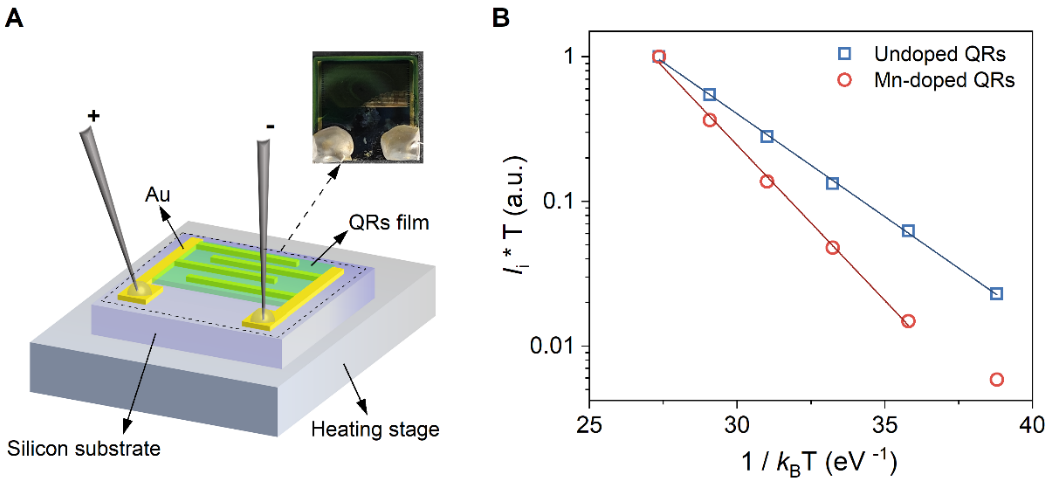

Fig. S19. Measurement of ionic migration activation energy. (A) Schematic diagram of the transport properties measurement setup for QR thin films. The inset in the top right corner is a photograph of the device. (B) Measured $I_i T$ as a function of $1/k_B T$ for undoped Br-rich (blue squares) and 3.5% Mn-doped Br-rich (red circles) QR films. T is temperature, I_i is current, and k_B is the Boltzmann constant. The solid fitting curves were obtained using a single exponential function. The fitted ionic migration activation energy (E_i) values are 0.14 eV and 0.22 eV for the undoped Br-rich and 3.5% Mn-doped QR films, respectively.

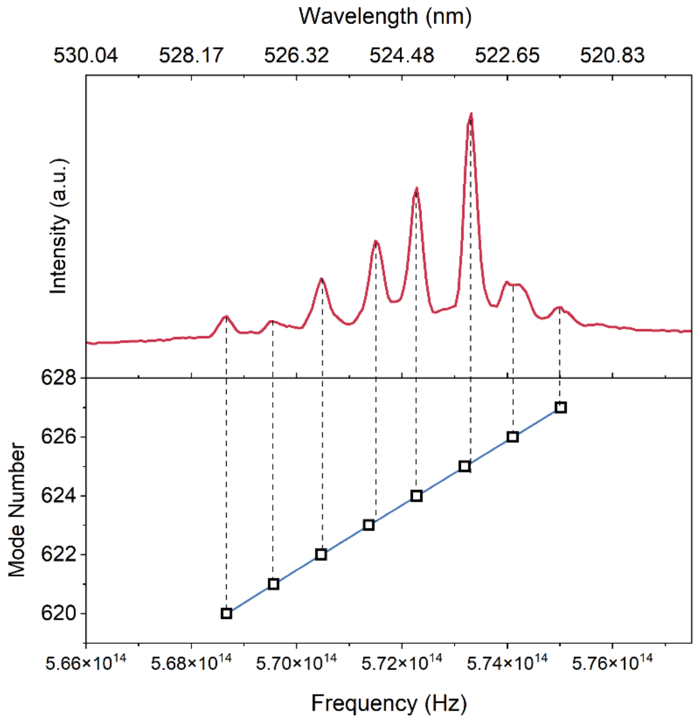

Fig. S20. Mode analysis of a whispering gallery mode (WGM) lasing spectrum with azimuthal mode numbers ranging from 620 to 627. The resonant condition in WGM resonators can be approximated by $2\pi nR = m\lambda$ ($m = 1, 2, 3, \ldots$), where n is the resonator's effective refractive index, R is the resonator's radius, m represents the number of wavelengths in a round trip through the cavity, and λ is the wavelength of the light(*57*). In our experiment, the resonator is a silica fiber with a refractive index of ~1.46. The free spectral range (Δv) can be calculated using $c/2\pi nR$. Our WGM calculations are consistent with the laser spectrum. Δv is calculated as ~9.07×10^{11} Hz, and the lasing mode spacing is ~0.83 nm at the lasing wavelength of 523.5 nm. The azimuth mode numbers m corresponding to the laser peak range from 620 to 627. These results demonstrate that our laser exhibits the characteristic features of WGM.

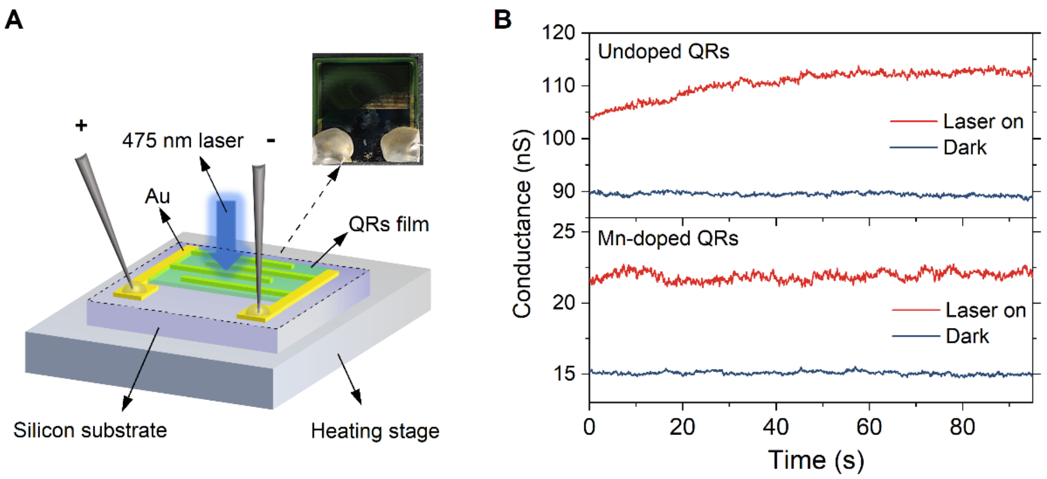

Fig. S21. Conductivity measurement for QR thin films. (A) Schematic diagram of the conductivity measurement setup for QR thin films. The inset in the top right corner is a photograph of the device. (B) Time-dependent conductance curves for undoped Br-rich (upper panel) and 3.5% Mn-doped (lower panel) QR films under dark conditions (blue) and 475 nm laser illumination (red).

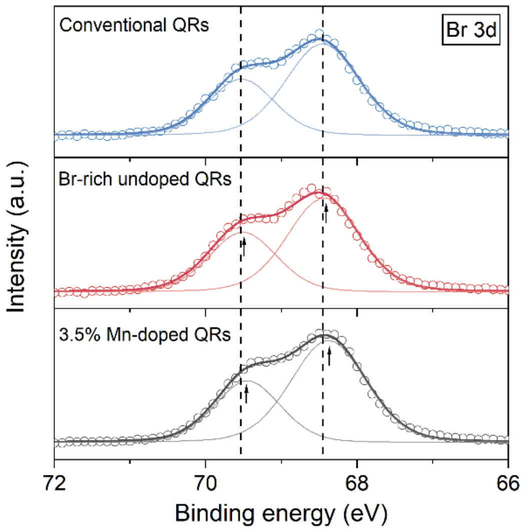

Fig. S22. X-ray photoelectron spectroscopy (XPS) spectra of Br 3d peaks for conventional (blue), Br-rich undoped (red), and 3.5% Mn-doped (black) QR films.

SUPPLEMENTARY NOTES

Supplementary Note 1. Modeling the transient lasing behavior of neutral and charged QDs and threshold estimation (modified gain-switching model)

Past studies have proposed the concept of zero-threshold optical gain(*24*). Considering an ideal bi-modal distribution of the average exciton number per quantum dot (QD) $\langle N \rangle$ and a uniform distribution of the average per-dot charge number $\langle n_c \rangle$, the optical-gain thresholds for neutral QDs, single-charged QDs and double-charged QDs are 1, 0.5, and 0, respectively(*24*). However, in real-life situations, it is commonly assumed that the distributions of the exciton number N and the charging number n_c follow Poisson statistics:

$$p(N) = e^{-\langle N \rangle} \langle N \rangle^{N} / N! \quad \text{(S1)}$$

$$q(n_c) = e^{-\langle n_c \rangle} \langle n_c \rangle^{n_c} / n_c! \quad \text{(S2)}$$

Then, for $\langle n_c \rangle$ = 0, 1, 2, 3, 4, 5, and 6, the calculated values for gain thresholds $\langle N_g \rangle$ are 1.15, 0.81, 0.46, 0.23, 0.10, 0.046, and 0.019, respectively.

The onset of the lasing regime is considerably more difficult than achieving optical gain. A crucial requirement is that the maximal rate of stimulated emission τ_0^{-1} outcompetes the optical-decay rate due to Auger recombination of the gain medium itself and photon losses in the cavity(*4*, *58*). To quantify the effect of Auger recombination on the lasing threshold $\langle N_{las} \rangle$ and estimate $\langle N_{las} \rangle$ at different $\langle n_c \rangle$, the transient lasing behavior of neutral and charged QRs has been studied using a model developed from the gain-switching model

with some modifications(*24*, *58*, *59*). The Poisson distribution of $\langle n_c \rangle$ and the biexciton gain in charged QDs with weak Auger recombination have been taken into account, and $\langle N_{las} \rangle$ has been calculated for $\langle n_c \rangle$ from 0 to 10 for the first time. The note is divided into two parts, each describing a different situation as follows.

Only single exciton gain occurs in charged QDs with severe Auger recombination:

To begin with, a Poisson distribution of the number of charges in the QD ensemble has been assumed in this model, as shown in Eq. S2. When the average number of charges per QD is $\langle n_c \rangle$, $q(n_c)$ is defined as the probability of finding a QD with n_c charges. In this case, neutral, single charged and multi-charged QDs (fig. S2) coexist in the QD ensemble with probability of $q(0)$, $q(1)$ and $(1-q(0)-q(1))$, respectively. The probability of absorption or stimulated emission per spin-allowed transition is denoted as γ. The "absorbing" transition from the charged exciton state ($|\mathrm{X}^-\rangle$ and $|\mathrm{X}^{n_c-}\rangle$) to the charged biexciton state ($|\mathrm{XX}^-\rangle$ and $|\mathrm{XX}^{n_c-}\rangle$) is blocked(*24*). Then, the coupled QD-light field system in the cavity can be described using the following set of kinetic equations:

Neutral QDs:

$$\frac{dP_0}{dt} = -2\gamma\phi P_0 + \frac{\gamma}{2}\phi P_\mathrm{X} + \frac{P_\mathrm{X}}{\tau_\mathrm{X}} \tag{S3}$$

$$\frac{dP_\mathrm{X}}{dt} = -2\frac{\gamma}{2}\phi P_\mathrm{X} + 2\gamma\phi P_0 + 2\gamma\phi P_\mathrm{XX} - \frac{P_\mathrm{X}}{\tau_\mathrm{X}} + \frac{P_\mathrm{XX}}{\tau_\mathrm{XX}} \tag{S4}$$

$$\frac{dP_\mathrm{XX}}{dt} = -2\gamma\phi P_\mathrm{XX} + \frac{\gamma}{2}\phi P_\mathrm{X} - \frac{P_\mathrm{XX}}{\tau_\mathrm{XX}} \tag{S5}$$

Constraint condition 1: $P_0 + P_X + P_{XX} = q(0)$

Single charged QDs:

$$\frac{dP_{0^-}}{dt} = -\gamma\phi P_{0^-} + \gamma\phi P_{X^-} + \frac{P_{X^-}}{\tau_{X^-}} \quad \text{(S6)}$$

$$\frac{dP_{X^-}}{dt} = -\gamma\phi P_{X^-} + \gamma\phi P_{0^-} - \frac{P_{X^-}}{\tau_{X^-}} \quad \text{(S7)}$$

Constraint condition 2: $P_{0^-} + P_{X^-} = q(1)$

Multi-charged QDs:

$$\frac{dP_{0^{n_c-}}}{dt} = \gamma\phi P_{X^{n_c-}} + \frac{P_{X^{n_c-}}}{\tau_{X^{n_c-}}} \quad \text{(S8)}$$

$$\frac{dP_{X^{n_c-}}}{dt} = -\gamma\phi P_{X^{n_c-}} - \frac{P_{X^{n_c-}}}{\tau_{X^{n_c-}}} \quad \text{(S9)}$$

Constraint condition 3: $P_{0^{n_c-}} + P_{X^{n_c-}} = 1 - q(0) - q(1)$

Total photon density in the cavity:

$$\frac{d\phi}{dt} = \gamma\phi n_{QD}(2P_{XX} + P_{X^-} + P_{X^{n_c-}} - 2P_0 - P_{0^-}) - \frac{\phi}{\tau_c} \quad \text{(S10)}$$

Therein, P_0, P_X and P_{XX} are the fractions of unexcited QDs, QDs containing a single exciton, and QDs containing at least 2 excitons, respectively. τ_X, τ_{XX}, and τ_c are the lifetimes of the single exciton (X), the biexciton (XX), and cavity photons, respectively. The upper-corner mark of 0, X, and XX in P and τ indicates the charge number contained in the QDs. n_{QD} is the QD density in the gain medium, and ϕ is the photon density in the cavity. The corresponding parameters are listed in Supplementary Table S1. To initiate the buildup of a photon population, one photon per unit volume (ϕ_0 = 1 cm^{-3}) is seeded in the cavity at time t = 0. Exciting the QDs with a short δ-function-like pulse, the temporal evolutions of fractions of each QD exciton

states and ϕ are obtained using Eqs. S3-S10, and three constraint conditions, as shown in fig. S3A-D.

Biexciton gain occurs in charged QDs with weak Auger recombination:

In general, it is difficult to populate the biexciton state in charged QDs, so the biexciton gain is usually ignored at low $\langle N \rangle$. However, it has been taken into consideration in QDs with weak Auger recombination. For example, in our QR system, significant biexciton emission is obtained even at a low excitation fluence of $\langle N \rangle$ = 0.02 (Fig. 2F). In this case, the "emitting" transition from the charged biexciton state ($|XX^{-}\rangle$ and $|XX^{n_c-}\rangle$) to the charged exciton state ($|X^{-}\rangle$ and $|X^{n_c-}\rangle$) is open, while the "absorbing" transition from the charged exciton state to the charged biexciton state remains blocked (fig. S2). The coupled QD-light-field system in the cavity can be described as follows:

Neutral QDs:

$$\frac{dP_0}{dt} = -2\gamma\phi P_0 + \frac{\gamma}{2}\phi P_X + \frac{P_X}{\tau_X} \tag{S11}$$

$$\frac{dP_X}{dt} = -2\frac{\gamma}{2}\phi P_X + 2\gamma\phi P_0 + 2\gamma\phi P_{XX} - \frac{P_X}{\tau_X} + \frac{P_{XX}}{\tau_{XX}} \tag{S12}$$

$$\frac{dP_{XX}}{dt} = -2\gamma\phi P_{XX} + \frac{\gamma}{2}\phi P_X - \frac{P_{XX}}{\tau_{XX}} \tag{S13}$$

Constraint condition 1: $P_0 + P_X + P_{XX} = q(0)$

Single charged QDs:

$$\frac{dP_{0^-}}{dt} = -\gamma\phi P_{0^-} + \gamma\phi P_{X^-} + \frac{P_{X^-}}{\tau_{X^-}} \tag{S14}$$

$$\frac{dP_{\mathrm{X}^-}}{dt} = -\gamma\phi P_{\mathrm{X}^-} + \gamma\phi P_{0^-} + 2\gamma\phi P_{\mathrm{XX}^-} - \frac{P_{\mathrm{X}^-}}{\tau_{\mathrm{X}^-}} + \frac{P_{\mathrm{XX}^-}}{\tau_{\mathrm{XX}^-}} \quad \text{(S15)}$$

$$\frac{dP_{\mathrm{XX}^-}}{dt} = -2\gamma\phi P_{\mathrm{XX}^-} - \frac{P_{\mathrm{XX}^-}}{\tau_{\mathrm{XX}^-}} \quad \text{(S16)}$$

Constraint condition 2: $P_{0^-} + P_{X^-} = q(1)$

Multi-charged QDs:

$$\frac{dP_{0^{n_c-}}}{dt} = \gamma\phi P_{\mathrm{X}^{n_c-}} + \frac{P_{\mathrm{X}^{n_c-}}}{\tau_{\mathrm{X}^{n_c-}}} \quad \text{(S17)}$$

$$\frac{dP_{\mathrm{X}^{n_c-}}}{dt} = -\gamma\phi P_{\mathrm{X}^{n_c-}} + 2\gamma\phi P_{\mathrm{XX}^{n_c-}} - \frac{P_{\mathrm{X}^{n_c-}}}{\tau_{\mathrm{X}^{n_c-}}} + \frac{P_{\mathrm{XX}^{n_c-}}}{\tau_{\mathrm{XX}^{n_c-}}} \quad \text{(S18)}$$

$$\frac{dP_{\mathrm{XX}^{n_c-}}}{dt} = -2\gamma\phi P_{\mathrm{XX}^{n_c-}} - \frac{P_{\mathrm{XX}^{n_c-}}}{\tau_{\mathrm{XX}^{n_c-}}} \quad \text{(S19)}$$

Constraint condition 3: $P_{0^{n_c-}} + P_{\mathrm{X}^{n_c-}} = 1 - q(0) - q(1)$

Total photon density in the cavity:

$$\frac{d\phi}{dt} = 2\gamma\phi n_{\mathrm{QD}}(P_{\mathrm{XX}} + P_{\mathrm{XX}^-} + P_{\mathrm{XX}^{n_c-}} - P_0) + \gamma\phi n_{\mathrm{QD}}(P_{\mathrm{X}^-} + P_{\mathrm{X}^{n_c-}} - P_{0^-}) - \frac{\phi}{\tau_c} \quad \text{(S20)}$$

Using the same method and parameters mentioned above, the temporal evolutions of fractions of each QD exciton states and ϕ were shown in fig. 3E-H. By integrating $\phi(t)$ from 0 to ∞, the total number of photons (Φ) emitted into the cavity per excitation pulse was calculated.

Finally, we modeled Φ as a function of $\langle N \rangle$ at different biexciton Auger lifetime $\tau_{\mathrm{A,XX}}$ (or $\langle n_c \rangle$) and demonstrated the dependence of $\langle N_{las} \rangle$ on $\tau_{\mathrm{A,XX}}$ (or $\langle n_c \rangle$) in fig. S4.

Supplementary Note 2. Quantum confinement of QRs with twofold degenerate band edge

According to recent research(*60*), large $CsPbBr_3$ perovskite nanocrystals exhibit quantum confinement when their volume does not exceed ~100^3 nm^3. The Auger recombination rate can decrease exponentially, and the emission of biexcitons can increase in large perovskite nanocrystals due to nonlocal effects. In our study, the dimensions of the QRs exceed the exciton Bohr radius of the bulk $CsPbBr_3$ material. However, the volume of the QRs is much smaller than 100^3 nm^3. Based on spectroscopic data, we confirm that QRs exhibit quantum confinement in the following aspects.

1) Blue shift of the PL spectrum. The QRs' PL peak center at 517 nm (Fig. 2D) is blue-shifted relative to the 2.36 eV (525 nm) bandgap of the bulk $CsPbBr_3$ material at room temperature(*61*). This indicates that quantum confinement increases the QRs' bandgap compared to the bulk material.

2) Broadening of the PL spectrum. The PL spectral linewidth of the QR ensemble in solution is ~83 meV (Fig. 2D), which is wider than the 70 meV observed in bulk $CsPbBr_3$ material(*61*). This broadening arises from inhomogeneity in the QR sample and is attributed to the quantum size effect of the QRs, specifically the quantum confinement effect.

3) Shorter biexciton lifetimes. The biexciton lifetime of the QRs is measured to be ~2 ns at the single-particle and ensemble levels (fig. S14E and Fig. 3A). Bulk

$CsPbBr_3$ material with a carrier density of 10^{18} cm^{-3} was predicted to have a biexciton lifetime of ~10 ns(*62*). The biexciton lifetime of the QRs is shorter than that of the bulk material. This suggests that the Coulomb scattering between carriers in QRs is still enhanced by the quantum confinement effect.

4) Photon statistics properties for identifying the quantum confinement effects in single QRs. For typical $CsPbBr_3$ quantum dots (QDs) with quantum confinement (~6 nm size), the $g^{(2)}(0)$ values in their second-order correlation function curves were much lower than 0.5(*63*), indicating strong antibunching emission caused by the quantum confinement effect. Therefore, the quantum confinement effect of QRs can be more intuitively reflected by second-order correlation function curves, i.e. photon statistics properties. This is because, due to the quantum confinement effect, there is only one emission center in a QR that mainly emits single exciton and biexciton photons. A time-gated technique can remove the biexciton emission and leave pure single exciton emission based on the shorter biexciton lifetime(*63–65*, *35*). The antibunching single exciton emission has a low $g^{(2)}(0)$ value in the second-order correlation function curve. For example, in this manuscript, the $g^{(2)}(0)$ value of single $CsPbBr_3$ QRs is very high (Fig. 2F, upper panel) due to a high biexciton quantum yield. According to the biexciton decay curve (fig. S15), a time gate of 4 ns was set to eliminate the emission of biexcitons. This results in a time-gated second-order correlation function with a low $g^{(2)}(0)$ value of 0.04 (fig. 2F, lower

panel). This low $g^{(2)}(0)$ value indicates strong antibunching behavior of single exciton emission in QRs, which firmly confirms the existence of quantum confinement and a single emission center. For bulk materials with a large number of emission centers, the $g^{(2)}(0)$ value is 1, with all emission centers contributing to it. The time-gating technique can only eliminate the contribution of biexcitons with shorter lifetimes. However, numerous exciton photons from different emission centers still contribute to the $g^{(2)}(0)$ value. Thus, the $g^{(2)}(0)$ value remains 1. Consequently, QRs exhibit entirely distinct photon statistics compared to bulk materials. Additionally, the radiative lifetime τ_r ratio of 2 between charged excitons and neutral excitons, as well as the ratio of 4 between neutral biexcitons and excitons, indicates a twofold degenerate asymmetric band structure model of QRs (fig. S6I and Supplementary Note 6).(*66*)

Supplementary Note 3. Indispensable role of CsBr-rich synthetic environments in generating the self-charging effect of QRs

A CsBr-rich synthetic environment is essential for synthesizing self-charged QRs. Conventional-synthesized QRs exhibit no self-charging effect without a CsBr-rich environment. This conclusion can be confirmed by comparing the spectra of conventional-synthesized QRs and CsBr-rich-synthesized QRs. First, the PL properties of single conventional-synthesized QRs are measured using single-dot PL spectroscopy. Two typical PL trajectories are shown in fig. S8. The first and last 40 s of the PL trajectories are marked with red and blue regions, respectively. The fluorescence lifetime-intensity distribution (FLID) maps of the two regions visually reveal the constant PL intensities and lifetimes, showing that the PL emission properties do not change over time. Fitting the decay curves yields the lifetimes of X^{n_c-} and XX^{n_c-} of ~ 12 ns and ~2.1 ns, respectively. These values are consistent with those of neutral (n_c = 0) CsBr-rich-synthesized QRs. These results demonstrate that conventional QRs remain neutral from beginning to end, indicating the absence of a self-charging effect. The indispensable role of CsBr-rich synthetic environments can be further substantiated by the weaker 1S excitonic absorption peak of CsBr-rich-synthesized QRs compared to conventional QRs (fig. S6C), which suggests the presence of extra charges (self-charging effect) within the CsBr-rich-synthesized QRs.

Supplementary Note 4. Determination of the excitation conditions $\langle N \rangle$ and the absorption cross-section σ_a in single QRs

The average exciton number per QD $\langle N \rangle$ can be calculated by:

$$I = b\left(1 - e^{-\langle N \rangle}\right) = b\left(1 - e^{-\alpha \cdot P}\right) \quad \text{(S21)}$$

The parameter α = 0.049 is obtained by fitting the PL saturation curves with Eq. S21, which records the correlation between the PL intensity (I) and the corresponding pump laser power density (*P*). In our single QR measurements, $\langle N \rangle$ = 0.2 can be obtained at a specific excitation power of 4.08 W/cm^2. j_{exc} is the excitation photon flux (photons × cm^{-2}) and can be calculated by $j_{exc} = P / \left(F \cdot h\nu\right)$, where F was 10 MHz in our system. Due to $\langle N \rangle = \sigma_a \cdot j_{exc}$, we can get the absorption cross-section σ_a *o*f the single QR as ~ 2.22×10^{-13} cm^2 (fig. S6D). Note that the multiexciton emission photons were removed from the PL saturation curve using the time-gating method(*66*) to ensure an accurate $\langle N \rangle$.

Supplementary Note 5. Determination of the radiative lifetimes τ_r of single exciton and biexciton

The radiative lifetimes τ_r of X and XX can be determined by:

$$\begin{cases} QY_{\mathrm{X}} = \dfrac{k_{r,\mathrm{X}}}{k_{r,\mathrm{X}} + k_{nr,\mathrm{X}}} = \dfrac{k_{r,\mathrm{X}}}{k_{\mathrm{X}}} = \dfrac{\tau_{\mathrm{X}}}{\tau_{r,\mathrm{X}}} \\ QY_{\mathrm{XX}} = \dfrac{k_{r,\mathrm{XX}}}{k_{r,\mathrm{XX}} + k_{nr,\mathrm{XX}}} = \dfrac{k_{r,\mathrm{XX}}}{k_{\mathrm{XX}}} = \dfrac{\tau_{\mathrm{XX}}}{\tau_{r,\mathrm{XX}}} \end{cases} \tag{S22}$$

Specifically, the second-order correlation function $g^{(2)}(\tau)$, as well as the decay curves of the single exciton and the biexciton at different PL intensity levels with an interval of 30 kcounts/s (schematically displayed in fig. S11), are extracted from the two PL regions in fig. S6E. For single exciton QY, we set the QY of the highest intensity level in the PL intensity time trajectory as a unity(*67*), so the QY of the other intensity levels can be expressed as $\mathrm{QY}_{\mathrm{X}} = I / I_{\max}$. For QY of biexciton, the QY of the various intensity levels is determined by $\mathrm{QY}_{\mathrm{XX}} = \mathrm{QY}_{X} \cdot g^{2}(0)$, where $g^{(2)}(0)$ value is obtained from the original $g^{(2)}(\tau)$ curve at $\tau = 0$. τ_{X} and τ_{XX} are obtained by mono-exponentially fitting the decay curves of single exciton and biexciton, respectively. Using Eq. S22, we can determine $\tau_{r,\mathrm{X}}$ and $\tau_{r,\mathrm{XX}}$ at different PL intensity for the two PL regions, as shown in fig. S6I.

Supplementary Note 6. Self-charging effect and reduced Auger recombination in single QRs

We demonstrate the reduced Auger recombination and unstable self-charging effect in undoped perovskite QRs. The weaker 1S excitonic absorption peak of CsBr-rich QRs compared to conventional QRs suggests the presence of extra charges (self-charging effect) within the CsBr-rich QRs (fig. S6C). We reveal the reduced Auger recombination and unstable self-charging effect in undoped perovskite QRs using single-dot PL spectroscopy. A typical PL intensity trajectory shows an increase in PL intensity over time (fig. S6E), which is contrary to the commonly reported decrease in intensity(*38*). The rationale behind this anomalous phenomenon will be elucidated later. The low $g^{(2)}(0)$ value of the time-gated second-order correlation function ($g^{(2)}$) curve proves that the PL originates from a single QR particle (fig. S6F). An extremely high $g^{(2)}(0)$ value of 0.98 observed in the original $g^{(2)}$ curve, which is significantly higher than that of the conventional single QD (~0.15)(*43*, *44*). The $g^{(2)}$ curves provide insight into the photon statistics properties of single QDs, and the $g^{(2)}(0)$ value indicates the probability of two-photon emission under a single excitation pulse(*43*, *64*, *65*). Therefore, the very high $g^{(2)}(0)$ values of QRs indicate efficient biexciton emission due to reduced Auger recombination in QRs. In this study, we designed perovskite QRs to significantly reduce Auger recombination via a state-engineering strategy (see Methods). We constructed the QR by elongating the QD in one dimension to form a rod-like structure with a higher

aspect ratio (Fig. 1C). This structure plays a critical role in suppressing Auger recombination. In this structure, exciton motion becomes more diffusive and their collisions diminish(*28*, *68*), the spatial overlap of exciton wave functions decreases, and the Coulomb scattering between carriers weakens(*30*, *69*). Consequently, the non-radiative Auger recombination of biexcitons in QRs decreases, thereby increasing the biexciton quantum yield. Thus, the $g^2(0)$ values of single QRs are very high.

The biexciton lifetimes for the two PL regions were obtained using the first-photon analysis method(*70*) (fig. S6G). Surprisingly, the biexciton lifetime increases from 0.89 ns (red region) to 2.33 ns (blue region). A significant prolongation is also observed in the single exciton lifetime (fig. S9). The FLID maps of the two regions in fig. S6H also visually reveal the considerable increase in the single exciton lifetime and slight increase in PL intensity (see fig. S10 for more examples). To further investigate this anomalous prolongation, the radiative lifetimes τ_r of single exciton and biexciton as a function of PL intensity for the two PL regions are obtained (fig. S6I, detailed in Supplementary Note 5). The τ_r ratios of the different excitons in the two PL regions can then be obtained from fig. S6I. The τ_r ratio of 4 between the single exciton and the biexciton in the blue region is consistent with that of neutral QDs(*47*), indicating that the QR is neutral in the blue region. In the red region, the τ_r ratio of 2 indicates that the QR is charged according to the twofold-degenerate asymmetric band structure model of QDs(*66*, *71*) (fig. S12). The

charged QR in the red region is further confirmed by the radiative lifetime ratios of 2 for single excitons and 1 for biexcitons between the red and blue PL regions. Thus, the prolonged lifetimes of single excitons and biexcitons can be attributed to the QR transforming from a charged state (red region) to a neutral state (blue region) with increasing excitation time. This also explains the anomalous increase in PL intensity in fig. S6E, because the PL intensity of a neutral QR is higher than that of a charged QR.

We construct a charging-associated Auger recombination model (Supplementary Note 7) to calculate the evolution of the charging number n_c and nonradiative Auger lifetime of biexciton ($\tau_{A,XX^{n_c-}}$) with time (fig. S6J). The results show that the QR initially possesses ~20 charges (red circles), demonstrating an excellent self-charging effect. However, under continuous illumination, these charges disappear due to the loss of hole acceptors. As n_c decreases, $\tau_{A,XX^{n_c-}}$ prolongs due to the reduction of Auger pathways (blue stars). Remarkably, our QRs exhibit an Auger lifetime of 13.9 ns for neutral XX (n_c = 0, blue solid star), which is two orders of magnitude longer than that of conventional CdSe-based QDs and perovskite QDs (30-300 ps)(*4*). The significantly reduced Auger recombination enables high PLQYs of charged excitons (90% in fig. S6E) and XX (98% in fig. S6F), as well as sufficient n_c in QRs. Both the self-charging effect and the suppressed Auger process of QRs contribute to achieving lasing with an ultra-low threshold. Therefore, stabilizing the hole acceptors and the resulting QR charging is essential.

Supplementary Note 7. Determination of charge number n_c and biexciton Auger lifetime $\tau_{\mathrm{A,XX}}$ in charged QRs

In a charged QR with electron number n_c, the recombination lifetimes of single exciton and biexciton (including radiative and nonradiative Auger recombination) differ from those in a neutral QR due to changes in the number of nonradiative Auger pathways for single exciton and biexciton. We construct a charging-associated Auger recombination model (fig. S12) and use Eq. S23 to calculate n_c.

$$\begin{cases} k_{\mathrm{X}^{n_c-}} = k_{r,\mathrm{X}^{n_c-}} + k_{nr,\mathrm{X}^{n_c-}} = 2k_{r,\mathrm{X}} + \dfrac{k_{\mathrm{A,XX}}}{8}\cdot 2n_c \\ k_{\mathrm{XX}^{n_c-}} = k_{r,\mathrm{XX}^{n_c-}} + k_{nr,\mathrm{XX}^{n_c-}} = 4k_{r,\mathrm{X}} + \dfrac{k_{\mathrm{A,XX}}}{8}\cdot 4\left(n_c+2\right) \end{cases}, \qquad \text{(S23)}$$

considering the radiative recombination rate ratios of different exciton species. Therein, $k_{\mathrm{X}^{n_c-}}$, $k_{r,\mathrm{X}^{n_c-}}$ and $k_{nr,\mathrm{X}^{n_c-}}$ represent the total recombination rate, the radiative recombination rate and the nonradiative recombination rate of single exciton in the charged QR with n_c charges, respectively. $k_{\mathrm{XX}^{n_c-}}$, $k_{r,\mathrm{XX}^{n_c-}}$ and $k_{nr,\mathrm{XX}^{n_c-}}$ represent the total recombination rate, the radiative recombination rate, and the nonradiative recombination rate of biexciton in the charged QR with n_c charges, respectively. $k_{r,\mathrm{X}}$ and $k_{\mathrm{A,XX}}$ represent the radiative recombination rate of single exciton and the Auger nonradiative recombination rate of biexciton in the neutral QR, respectively. $\tau_{\mathrm{X}^{n_c-}}$ and $\tau_{\mathrm{XX}^{n_c-}}$ can be obtained by fitting the decay curves of the single exciton and the biexciton, which were extracted from the bright state of the PL intensity trajectory with each 10 s time bin. Extracting the bright state ensures that additional nonradiative

processes, such as surface state-induced nonradiative recombination, do not need to be considered in our calculations. Then $k_{r,\mathrm{X}}$ equals to the total recombination rate k_{X}, the reciprocal of τ_{X}. For the undoped QRs, τ_{X} was obtained by fitting the decay curves of single excitons extracted from the bright state of the PL intensity trajectory after 190 s. For Mn-doped QRs, τ_{X} was set to 11.1 ns, which is the same as the mean value of τ_{X} for undoped QRs (fig. S9), since the QRs' size and shape were unchanged before and after doping (fig. S13). Then, n_c and $\tau_{\mathrm{A,XX}}$ can be obtained simultaneously.

Supplementary Note 8. Estimation of the lasing threshold for electrical-pumped lasers

The estimation of the lasing threshold for electrical-pumped lasers is based on the three-state model in Reference (*72*), with modifications considering that the QRs are charged rather than neutral. All cases are listed below for completeness. The corresponding models are displayed in fig. S2. The ground state $|0\rangle$, single exciton state $|\mathrm{X}\rangle$, and biexciton state $|\mathrm{XX}\rangle$ occur with probabilities P_0, P_X, and P_XX, respectively. The excitation rate of each QR is g.

Neutral QRs:

Under steady-state conditions, the balance between upward and downward optical transitions can be written as follows:

$$\begin{cases} P_\mathrm{XX}/\tau_\mathrm{XX} = gP_\mathrm{X} \\ P_\mathrm{X}/\tau_\mathrm{X} = gP_0 \end{cases} \tag{S24}$$

Constraint condition: $P_0 + P_\mathrm{X} + P_\mathrm{XX} = 1$

Therein, τ_X and τ_XX represent the lifetimes of the single exciton and the biexciton, respectively. Since single excitons do not contribute to optical gain, the net optical gain G is equal to $G_0(P_\mathrm{XX} - P_0)$. G_0 is the saturated gain achieved when all QRs have biexcitons. The optical-gain threshold can then be obtained from $P_\mathrm{XX} = P_0$, and the lasing threshold can be obtained from $P_\mathrm{XX} - P_0 = 0.5$ by assuming that the attainment of the lasing regime requires $G = 0.5G_0$ (*72*). According to eq. S24, the optical-gain threshold and the lasing threshold in terms of g can be calculated as follows:

$$g_{th,gain} = \frac{1}{\sqrt{\tau_X \tau_{XX}}}$$
$$g_{th,las} = \frac{1+\sqrt{1+12\,\tau_{XX}/\tau_X}}{2\tau_{XX}} \quad \text{(S25)}$$

The excitation rate *g* can be further expressed as $g = \sigma_e\left(j/e\right)$, where the QD electrical cross-section σ_e equals to σ_g / f. σ_e is the geometrical cross-section of QDs, and *f* is the areal semiconductor filling factor of QD ensemble, taking 0.5 as usual. *j* is the current density, and *e* represents the elementary charge. The optical-gain threshold and the lasing threshold in terms of *j* can then be calculated as follows:

$$j_{th,gain} = \frac{ef}{\sigma_g \sqrt{\tau_X \tau_{XX}}}$$
$$j_{th,las} = \frac{ef(1+\sqrt{1+12\,\tau_{XX}/\tau_X})}{2\sigma_g \tau_{XX}} \quad \text{(S26)}$$

Taking σ_g = 14×32 nm^2, τ_X = 11.37 ns and τ_{XX} = 2.36 ns (Supplementary Table S1), we get the lasing threshold $j_{th,las}$ of 10.85 A/cm^2.

Single charged QRs:

Under steady-state conditions, although biexcitons exist in our charged QR system, the rate of the transition from the single exciton state to the biexciton state should be much less than *g* because it is suppressed. Therefore, we do not include biexcitons in our conservative estimation. The balance between upward and downward optical transitions can then be written as follows:

$$P_{X^-} / \tau_{X^-} = gP_{0^-} \quad \text{(S27)}$$

Constraint condition: $P_{0^-} + P_{X^-} = 1$

In the equation, τ_{X^-} represents the lifetime of single exciton in charged QRs. The optical-gain threshold can be obtained from $P_{X^-} = P_{0^-}$, and the lasing threshold can be obtained from $P_{X^-} - P_{0^-} = 0.5$. According to S27, the optical-gain threshold and the lasing threshold can be calculated in terms of g as follows:

$$
\begin{aligned}
g_{th,gain^-} &= \frac{1}{\tau_{X^-}} \\
g_{th,las^-} &= \frac{3}{\tau_{X^-}}
\end{aligned}
\tag{S28}
$$

The lasing threshold in terms of j can then be calculated as follows:

$$
\begin{aligned}
j_{th,gain^-} &= \frac{ef}{\sigma_g \tau_{X^-}} \\
j_{th,las^-} &= \frac{3ef}{\sigma_g \tau_{X^-}}
\end{aligned}
\tag{S29}
$$

Taking σ_g = 14×32 nm^2 and $\tau_{X^-} = 1/(k_{r,X^-} + k_{A,X^-}) = 1/(2k_{r,X} + 1/4k_{A,XX})$ = 5.15 ns, we get the lasing threshold j_{th,las^-} of 10.4 A/cm^2.

Multi-charged QRs:

Under steady-state conditions, the conservative estimation does not take into consideration biexcitons. The balance between upward and downward optical transitions can then be written as follows:

$$
P_{X^{n_c*}} / \tau_{X^{n_c*}} = g P_{0^{n_c*}} \tag{S30}
$$

Constraint condition: $P_{0^{n_c*}} + P_{X^{n_c*}} = 1$

Therein, $\tau_{X^{n_c*}}$ represents the lifetime of single exciton in multi-charged QRs.

The optical-gain threshold can be obtained from $P_{X^{n_c}*}=0$, and the lasing threshold can be obtained from $P_{X^{n_c}*}=0.5$. According to S30, the optical-gain threshold and the lasing threshold in terms of g can be calculated as follows:

$$g_{th,gain^{n_c}*}=0$$
$$g_{th,las^{n_c}*}=\frac{1}{\tau_{X^{n_c}*}} \tag{S31}$$

The lasing threshold in terms of j can then be calculated as follows:

$$j_{th,gain*}=0$$
$$j_{th,las*}=\frac{ef}{\sigma_g \tau_{X^{n_c}*}} \tag{S32}$$

Taking σ_g = 14×32 nm^2 and $\tau_{\mathrm{X}^-}=1/(k_{r,\mathrm{X}^{n_c-}}+k_{\mathrm{A},\mathrm{X}^{n_c-}})=1/(2k_{r,\mathrm{X}}+4k_{\mathrm{A}})$ = 4.72 ns, we get the lasing threshold $j_{th,las*}$ of 3.79 A/cm^2.

Supplementary Note 9. Estimation of the lasing threshold $\langle N_{th} \rangle$ for nanosecond and femtosecond pulses pumping

Nanosecond pulses pumping:

When excited by an excitation source, the probability of finding a QR in the N-exciton state follows a Poisson distribution:

$$P(N,\langle N\rangle) = \langle N\rangle^{N} e^{-\langle N\rangle} / N! \tag{S33}$$

Setting the QY of a QR with m excitons to be $Q_{m\mathrm{X}}$, the total intensity of QR ensemble I_{total} can be expressed as:

$$I_{total} = \sum_{N=1}^{\infty} P(N,\langle N\rangle) \sum_{m=1}^{\infty} Q_{m\mathrm{X}} \tag{S34}$$

In the case of $Q_{m\mathrm{X}}(m>1)=0$, only single exciton emission exists in the QR ensemble, and I_{total} can be simplified to $I_{total} = I_{\mathrm{X}} = (1-P(0))\cdot Q_{\mathrm{X}} = 1-e^{\langle N\rangle}$, considering the QY of the single exciton to be unity; In the case of $Q_{m\mathrm{X}}(m>1)\neq 0$, single exciton and multiexciton ($m \geq 2$) contribute to the QR emission. $Q_{m\mathrm{X}}$ can be obtained by considering the corresponding radiative lifetime $k_{r,m\mathrm{X}}$ and non-radiative lifetime $k_{nr,m\mathrm{X}}$ of the multiexciton:

$$\begin{cases} k_{r,m\mathrm{X}} = \left[(m-2)^2+4\right]\cdot \dfrac{k_{r,\mathrm{XX}}}{4} \\ k_{nr,m\mathrm{X}} = \left[(m-2)^2+4\right]\cdot(2m+n_c-2)\cdot \dfrac{k_{\mathrm{A,XX}}}{8} \\ Q_{m\mathrm{X}} = \dfrac{k_{r,m\mathrm{X}}}{k_{r,m\mathrm{X}}+k_{nr,m\mathrm{X}}} \end{cases} \tag{S34}$$

Then the expression of I_{total} can then be summarized as follows:

When $m \leq 2$ (single exciton only):

$$I_{total} = I_{\mathrm{X}} = [1 - P(0)] \cdot 1 \tag{S35}$$

When $m \geq 2$ (a multiexciton-correlated PL saturation model):

$$I_{total} = I_{\mathrm{X}} + I_{m\mathrm{X}} = [1 - P(0)] \cdot 1 + [1 - P(0) - P(1)] \sum_{m=2}^{\infty} Q_{m\mathrm{X}} \tag{S36}$$

When $k_{\mathrm{A,XX}} = 0$ (Auger-free):

$$I_{total} = I_{Auger-free} = [1 - P(0)] \cdot 1 + [1 - P(0) - P(1)] \cdot \left(N - 2 + 1\right) \tag{S37}$$

To determine the correspondence between $\langle N \rangle$ and pump fluence *F,* we introduce the parameter *α*:

$$\langle N \rangle = \alpha \cdot F \tag{S38}$$

The parameter *α* is then determined to be 0.01 by fitting the dependence of the pump fluence on the PL intensity of QRs in fig. S16D with Eq. S36, combining Eqs. S33, S34 and S38. Therefore, $\langle N \rangle = 0.01 \cdot F$, yielding the corresponding $\langle N_{las} \rangle$ of 0.098. The curves of single-exciton-only PL intensity and Auger-free PL intensity are modeled using the aforementioned fitting parameter.

Femtosecond pulses pumping:

The correlation between pump fluence and $\langle N \rangle$ is determined by the pump fluence-dependent ground-state bleaching signal in transient absorption (TA) measurement. The magnitude of the ground-state bleaching signal after

complete multiexciton decay can be represented by a Poisson statistics model after ignoring higher-order terms: $\Delta A^{X} = A_0^X\left(1-e^{-\langle N\rangle}\right) = A_0^X\left(1-e^{-\alpha\cdot F}\right)$. Parameter α can be obtained as 0.064 by exponential fitting. Due to the limitations of the measurement conditions, the TA measurement was performed under 343 nm excitation. Since α is proportional to the absorption cross-section, α (400 nm excitation) is 0.47 times that of α (343 nm excitation) by considering the same ratio of absorption section in the steady-state absorption spectrum (fig. S13). Therefore, α (400 nm excitation) is 0.030. The absorption cross-section of the QR solution was obtained to be ~1.49×10^{-14} cm^2. According to $\langle N\rangle = 0.03\cdot F$, the threshold $\langle N\rangle$ value was found to be 0.087 under the femtosecond laser pumping.

Supplementary Note 10. Measuring the Auger lifetime of QRs via TA spectroscopy

Evaluating Auger recombination in QR material is crucial to this study. One important method for studying defect-related Auger recombination processes is excitation-density-dependent femtosecond transient absorption spectroscopy (fs-TAS) (*73*). The defect-state-assisted Auger recombination coefficient can be obtained by fitting the excitation-dependent delay curves of the photoinduced absorption (PA) signals with a triple-exponential function. We employed the fs-TAS to analyze the Auger recombination of QR material under different excitation densities. The resulting excitation-dependent PA signals are presented in fig. S18. The PA signals experience a rapid delay within a few picoseconds, approaching the relaxation time of hot carriers. However, due to the rapid relaxation of the PA signals, we were unable to extract a valid defect-state-assisted Auger recombination coefficient from the data.

To extract the valid defect-state-assisted Auger recombination coefficient from the PA signals of the materials, the materials must possess abundant, infinitely degenerate defect states and the carriers trapped within these states must undergo transitions(*73*). Therefore, the failure to obtain defect-state-assisted Auger recombination coefficient may be attributed to the following reasons. First, perovskite QRs do not possess abundant defect states due to the inherent defect tolerance of perovskite materials. Consequently, the defect states do not exhibit infinite degeneracy, making it impossible to form detectable excited-state

absorption. Second, perovskite QRs do not exhibit significant transitions of carriers trapped in defect states. In other words, the trapped carriers within QRs are not effectively re-excited to the excited state or converted into hot carriers by the excitation pulses. This is demonstrated by QRs' ability to maintain long-term charging by constructing stable trapping sites. Third, the rapid relaxation of the PA signals observed in our QR experiments should be associated with Coulomb multiparticle interactions, such as the biexciton effect(*74*). This well-recognized mechanism has been widely reported and can explain the rapid relaxation of the PA signals.

We employed fs-TAS to quantify the Auger recombination in QR material by analyzing the excitation-dependent ground-state bleaching signals(*75*). This well-established and reliable method directly determines Auger recombination rates through precise measurement of the biexciton lifetime. The ground-state bleaching signals were measured under low ($\langle N \rangle_0 = 0.11$, red circles) and high ($\langle N \rangle_1 = 1.44$, blue circles) excitation conditions, as shown in Fig. 3A. We observed that the TA kinetics under high excitation exhibited a distinct fast component compared to that under low excitation. We normalized the TA kinetics to their long-lived tails. The biexciton kinetic curve (inset) was obtained by subtracting the TA kinetics of $\langle N \rangle_0 = 0.11$ from that of $\langle N \rangle_1 = 1.12$. A single-exponential fit yielded a biexciton lifetime (τ_{XX}) of 1.89 ns, which is similar to the ~2 ns biexciton lifetime observed in single QR measurements (fig. S15). The single exciton lifetime (τ_X) was found to be 9.33

ns by fitting the long-lifetime region of the TA decay curve under low excitation condition with a single-exponential function. Using the formula $k_{A,XX} = k_{XX} - k_{r,XX} = 1/\tau_{XX} - 4k_{r,X} = 1/\tau_{XX} - 4/\tau_X$, where $k_{r,XX}$ and $k_{r,XX}$ are the radiative recombination rates of the single exciton and the biexciton, respectively, the non-radiative Auger recombination rate of the biexciton ($k_{A,XX}$) can be calculated to be 0.091 ns^{-1}. Therefore, the biexciton Auger lifetime ($\tau_{A,XX}$) of QRs is 11 ns ($\tau_{A,XX} = 1/ k_{A,XX}$), which is approximately two orders of magnitude longer than that of conventional CdSe-based QDs and perovskite QDs (30 - 300 ps).

In addition, single QD spectroscopy is a reliable and widely used method for obtaining biexciton lifetimes and non-radiative Auger rates. Extracting the photons from the bright state of the PL trajectories of single QRs can effectively avoid the influence of defect-induced non-radiative recombination on the measurements of biexciton lifetimes(*8*, *76*). In our work, we obtained single exciton lifetime (τ_X) from the PL decay curve of the bright-state photon and obtained biexciton lifetime (τ_{XX}) from the two-photon events in the bright-state of PL trajectories using the first-photon analysis method(*77*). Through the formulas $k_{A,XX} = k_{XX} - k_{r,XX} = 1/\tau_{XX} - 4k_{r,X} = 1/\tau_{XX} - 4/\tau_X$, we can obtain the non-radiative Auger rate and lifetime for the neutral biexciton as 0.072 ns^{-1} and 13.9 ns, respectively. Therefore, the biexciton Auger lifetime of QRs is approximately two orders of magnitude longer than that of conventional CdSe-based QDs and perovskite QDs (30-300 ps).

Supplementary Note 11. Measurement of Br ion migration activation energy

An increase in the Br ion migration energy barrier can be demonstrated by measuring the ionic migration activation energy (E_i). To obtain E_i, we measured the transport properties of undoped and 3.5% Mn-doped $CsPbBr_3$ QR films as a function of temperature (T). The thin QR films were deposited on a silicon substrate patterned with 50 µm-apart Cr/Au interdigitated electrodes, as shown in fig. S19A. An external electric field with a magnitude of 5 V was applied between the two electrodes, and the electric current was detected using a source measure unit under dark conditions.

It is generally accepted that $CsPbBr_3$ films only have ionic and electronic currents. Within our temperature range of 25 °C to 150 °C, ionic transport significantly contributes to the current (I_i) due to thermally activated ionic migration. The I_i and T can be determined using the standard Arrhenius model(*78*):

$$I_i \cdot T = A_i \exp\left(-E_i / k_B T\right), \quad \text{(S39)}$$

where A_i is the pre-exponential factor and k_B is the Boltzmann constant.

Figure S19b shows the measured $I_i T$ as a function of $1/k_B T$ for undoped (blue squares) and 3.5% Mn-doped (red circles) QR films. The $I_i T$ values increase as $1/k_B T$ decreases, indicating a significant contribution of thermally activated ionic migration to I_i. Through monoexponentially fitting, E_i values of 0.14 eV and 0.22 eV were obtained for the undoped and 3.5% Mn-doped QRs,

respectively. The larger E_i value for the 3.5% Mn-doped QRs suggests that hybridization between the Mn 3d orbitals and the Pb 6s-Br 4p antibonding states increases the energy barrier for Br^- migration.

Supplementary Note 12. Inductively coupled plasma mass spectrometry (ICP-MS) measurements

Sample pretreatment procedures: The purified Mn-doped perovskite QRs were washed with ethyl acetate and subjected to centrifugation and precipitation to remove residual solvents and ligands. Then, 100 μL of the purified QD solution was aliquoted and digested in 200 μL of concentrated HNO_3 until a clear solution was obtained. After complete digestion, the solution was diluted 10-, 100-, 1000-, 10,000-, and 100,000-fold with ultrapure deionized water to establish a concentration gradient for the ICP-MS measurement.

Calibration standards: The calibration standards were prepared from commercial, single-element stock solutions of Mn and Pb (PerkinElmer, USA). The 1000 ppm Mn standard solution was diluted in steps with ultrapure deionized water to create a concentration gradient of 1-100 ppb, which covers the expected Mn concentration in the digested QR solution. Similarly, the 1000 ppm Pb standard solution was diluted in steps to create a concentration gradient of 10-1000 ppb with ultrapure deionized water for calibration.

To correct for potential instrumental drift and matrix effects, an internal standard was added to each sample and standard solution. Throughout the analysis, the signal intensity of the internal standard was monitored, and all sample results were normalized using the internal standard response. A dilute acid blank was measured to rule out background contamination.

Measurement reproducibility: The inductively coupled plasma mass

spectrometry (ICP-MS) measurements were performed using a NexION 350X (PerkinElmer, USA). The reproducibility of the ICP-MS measurements was evaluated by analyzing each sample in triplicate. The relative standard deviation (RSD) was calculated for each element, and the RSD values were consistently below 4% for all analytes, indicating good measurement precision. Based on the measurement results, we calculated the atomic ratio of Mn (atomic %, with respect to Pb) to represent the doping percentage, as shown in Table S2.

Supplementary Note 13. Verification of the self-charging effect through conductivity measurements

We verified the self-charging effect of QRs through conductivity measurements. The self-charging effect in QRs originates from the introduction of bromine (Br_i^-) interstitial states acting as hole acceptors (Fig. 1D). Holes from excitons are captured by these Br_i^- interstitial states, leaving extra electrons in the conduction band of QRs (fig. S5B, left), which leads to self-charging effect. In undoped QRs, the Br_i^- interstitial states are unstable due to a tendency for self-reorganization. They migrate naturally to the QR surface and combine with Cs^+, resulting in the eventual loss of hole acceptors. As hole acceptors are gradually eliminated, the captured holes are progressively released. During electrical measurements, the migration of these Br_i^- interstitial states under laser illumination and the subsequent release of captured holes contribute to charge transport, leading to a gradual increase in photoconductivity over time.

In Mn-doped QRs, however, the increased energy barrier induced by Mn doping suppresses the migration of Br_i^- interstitial states, allowing the hole acceptors to persist and thus stabilizing QR charging. Consequently, Mn-doped QR films are expected to exhibit lower and more stable conductivity compared to undoped QR films.

Based on this analysis, we fabricated a device to measure electrical conductivity. As shown in fig. S21A, a thin QR film was deposited on a silicon substrate patterned with Cr/Au interdigitated electrodes (50 μm spacing). A bias

of 1 V was applied between the electrodes, and the current was monitored using a source measure unit under dark conditions and under laser illumination. The QRs were charged using a continuous-wave 475 nm laser.

The time-dependent conductance curves are presented in fig. S21B. When the undoped QR film was illuminated with the 475 nm laser, carriers were excited, resulting in a significant increase in conductance from 90 nS in the dark to 105 nS (fig. S21B, upper panel). In contrast, the Mn-doped QR film exhibited a much smaller increase in conductance under illumination, rising from 15 nS in the dark to 22 nS (fig. S21B, lower panel). This suggests that Mn doping impedes the mobility of Br ions in the QR films. Furthermore, the photoconductance of the undoped QR film continued to increase with illumination time until it reached saturation at around 60 s, whereas the photoconductance of the Mn-doped film remained relatively stable. These results suggest that Br_i^- states in undoped QR films migrate over time, which is consistent with the time-dependent increase in PL intensity observed in fig. S7. This implies that undoped QRs undergo continuous spontaneous discharge due to the gradual elimination of hole acceptors (Br_i^- interstitial states), while Mn-doped QRs can maintain stable self-charging.

Supplementary Note 14. Discussion on further reducing the laser threshold

To better guide experimental research, we theoretically calculated the dependence of the lasing threshold on Auger recombination (characterized by the biexciton Auger lifetime $\tau_{\mathrm{A,XX}}$) and charging number $\langle n_c \rangle$ based on a modified gain-switching model (see note S1). The results shown in Fig. 1B reveal the relationship between the maximum achievable lasing threshold and the Auger recombination and charging number. More quantitative results are shown in fig. S4C. As can be seen from the figures, the high charging number and the effective suppression of Auger recombination in QRs must be achieved simultaneously to lower the lasing threshold.

In our study, the Auger lifetime $\tau_{\mathrm{A,XX}}$ of QRs is 13.9 ns (Fig. 1F). As can be seen in fig. S4C, when charging number is smaller than 6, the curve ($\tau_{\mathrm{A,XX}}$ = 13.9 ns) is already very close to the theoretical curve ($\tau_{\mathrm{A,XX}} = \infty$). In this case, further suppression of the Auger recombination has little effect on reducing the laser threshold. When the charging number increases from 6 to 8, the lasing threshold $\langle N_{las} \rangle$ reduces from 0.04 to 0.02. Achieving stable 8-charge charging requires a higher Mn-doping concentration of 6.4% (fig. S14). However, the 6.4% Mn doping can introduce defects to reduce the PL intensity of QRs (Fig. 2H). Therefore, other methods need to be developed to stabilize the QR charging. When the charging number reaches 8, the laser threshold cannot be reduced to 0.01 even by arbitrarily extending the QR length to arbitrarily decrease the Auger recombination. However, extending the QR length comes at the cost

of introducing more defects and sacrificing QR performance. Therefore, lowering the laser threshold from the current level requires comprehensive consideration of multiple factors and require considerable effort.

Supplementary Note 15. A discussion on the similarities and differences with other charging approaches

Previous charge doping approaches, such as external potential control (electrochemical methods) and photochemical reactions(*25*, *79*, *80*), face several key limitations. First, the charging number introduced is low, often limited to only two in each QD. Second, QD charging is unstable and requires continuous operation to prevent charge loss from spontaneous discharge. Third, previous charging approaches involve external chemical operations that can damage the QD gain material(*25*, *80*–*82*). In our study, first, we elongated the QDs to construct quantum rods (QRs), which weaken exciton interactions. These rod-shaped structures can accommodate up to six charges. Second, we designed QRs with a self-charging effect by introducing bromine interstitial states as hole acceptors. We developed a Mn-doping strategy to stabilize the hole acceptors and achieve stable six-charge charging. Third, stable six-charge charging is achieved solely through the design of the gain material itself. This does not involve any external chemical operations during charging, so the gain material is not damaged. Using this method, we achieved a sub-one-tenth exciton lasing threshold.

A discussion of the similarities and differences with other elemental doping strategies

Compared to other transition metal elements with empty d-orbitals, Mn has the lowest electronegativity(*83*), which leads to strong long-range lattice stabilization and

optimal suppression of ionic migration. Mn^{2+} shares the same valence state as Pb^{2+}, enabling isovalent substitution at the Pb site without disrupting the original crystal structure of $CsPbBr_3$ perovskites. Importantly, Mn doping neither introduces intraband defect states, which destroy defect tolerance, nor alters the band edge energy level structure in quantum confined QRs, thereby ensuring stable optoelectronic performance(*83*). Additionally, due to their small atomic size, Mn doping can be achieved through a simple post-doping process (cation exchange method), which is more convenient than the synthesis routes required for many other dopants and fully compatible with our water-oil interface reaction strategy. Consequently, the Mn-doping approach is our optimal strategy.

The characteristics of other common elemental doping strategies in perovskite QDs are briefly summarized as follows:

Rare earth elements: Although rare earth doping can modulate the optical properties of perovskites, it typically relies on the hot-injection method, which is incompatible with our water-oil interface reaction system. Additionally, rare earth ions tend to introduce defect energy levels within the band gap due to their relatively large atomic size(*83*).

Sn: As a common isovalent dopant, Sn does not shift the absorption peak or alter the bromine band edge energy level structure. However, it is thermodynamically unstable and prone to oxidation, which significantly degrades the optical performance of doped materials. This unavoidable drawback limits its practical application(*83*).

Other transition metals (Ni, Zn, and Ag): Literature reports indicate that Ni and Zn can improve the properties of lead halide perovskites via Pb-site substitution. However, compared to Mn, they lack the synergistic advantages of strong lattice stabilization, a simple doping process, and uncompromised PL quantum yield(*78*). For example, some elements may introduce subtle lattice distortions or necessitate harsher doping conditions, making Mn a more balanced and reliable option(*83*).

Ag^+ undergoes heterovalent substitution with Pb^{2+}, forming a double perovskite structure ($A_2B'B''X_6$). This doping significantly improves conductivity and charge-carrier mobility (by nearly three orders of magnitude) and induces p-type characteristics, making it suitable for optoelectronic devices. However, it alters the intrinsic electronic structure of perovskites(*83*). In contrast, Mn's isovalent substitution maintains the original material properties while enhancing stability. This is more consistent with the design goal of optimizing the self-charging and lasing performance of QRs.

Supplementary Note 16. Experimental evidence for the presence of Br_i^- interstitial states

X-ray photoelectron spectroscopy (XPS) is a powerful and well-established analytical technique for determining the chemical state of elements within materials. It can also help us to identify the bonding environment of Br atoms within QRs. As shown in fig. S22, fine XPS spectra of the Br 3d region are presented for three types of QRs: conventional (blue line), Br-rich undoped (red line), and 3.5% Mn-doped (black line). These spectra can be assigned to Pb–Br lattice bonds. Fitting the doublet peaks reveals the following: For the conventional QRs, the binding energy peaks are located at 68.46 eV and 69.53 eV (upper panel). For the Br-rich undoped QRs (added with CsBr), the binding energy peaks shift slightly towards lower energies, to 68.43 and 69.50 eV respectively (middle panel). This decrease in binding energy is attributed to the enhanced electron-donating ability of the Br atoms, indicating the presence of Br ions in a different local coordination environment. For the 3.5% Mn-doped QRs (added with $MnBr_2$), the binding energy peaks shifted further to 68.38 and 69.45 eV (lower panel), suggesting a substantial increase in the number of Br ions. These Br ions are likely to be present as Br_i^- states or weakly bound surface Br^- species.

Additionally, the XPS spectra show Br/Pb ratios of 3.3 and 3.6 for the Br-rich undoped and 3.5% Mn-doped QRs, respectively. Br/Pb ratios greater than 3 indicate a

Br-rich environment in the QRs, consistent with previous reports on $CsPbBr_3$ nanocrystals synthesized under excess bromide conditions, which are associated with the formation of Br_i^- states(*84*).

However, the existing literature offers little direct experimental evidence of Br_i^- interstitial states (specific point defects)(*85*). Distinguishing Br_i^- states from other Br-rich species, such as adsorbed halides, Br-rich ligands, or antisite defects, by chemical analysis alone remains highly challenging. To further support the experimental findings, we refer to first-principles calculations from earlier studies(*86*, *87*). These calculations demonstrate that under Br-rich conditions, the formation energy of Br_i^- states is extremely low and that Br_i^- states are the primary intrinsic defects in $CsPbBr_3$ nanocrystals(*34*, *88*). This theoretical framework aligns well with our experimental results, providing additional support for the presence of Br_i^- interstitial states in the QRs.

Supplementary Note 17. Discussion of the anomalous increase in PL intensity of single QRs

The anomalous increase in PL intensity that we observed under light illumination differs from previous reports. First, ligand and defect rearrangement of QDs under illumination can lead to the gradual enhancement of PL intensity(*89*, *90*). In this case, the surface ligands and defects undergo reorganization upon light exposure without the involvement of photochemical reactions between the QDs and the surrounding environment. This rearrangement can result in more efficient passivation of surface defects or removal of poorly bound ligands, improving the QDs' electronic environment. The defect rearrangement phenomenon in $CsPbBr_3$ QDs reported in Ref. 46 differs from the photoinduced bromide ion migration and discharging mechanism described in our work. There is a notable difference: Ref. 46 reported PL enhancement in Br-deficient QDs over a few hours, whereas we observed an increase in PL intensity in Br-rich QRs within a few minutes.

Furthermore, an abnormal enhancement of PL intensity can be induced by photochemical reactions between QDs and surrounding gas molecules. Water molecules can adsorb onto the surface of QDs and interact with surface unsaturated bonds. This effectively passivates these trap states and reduces non-radiative recombination channels. This leads to an increase in PL intensity over time when QDs are exposed to continuous illumination(*91–94*). Oxygen molecules can also passivate QD surface defects, leading to an increase in the PL intensity(*95*, *96*). Additionally, oxygen molecules can convert photoinduced trion states with low

quantum yield into single-exciton states with high quantum yield via deionization under illumination(*97*, *98*). Photochemical reactions between QDs and the surrounding medium and ligands under illumination can also passivate surface defects and enhance PL intensity(*99–101*). Our single-QD measurements were conducted under a nitrogen atmosphere, which eliminates interference from photochemical reactions between QDs and surrounding gas molecules.

Our single-QD measurements were conducted under a nitrogen atmosphere, which eliminates interference from photochemical reactions between QDs and surrounding gas molecules. In our work, the anomalously increased PL intensity is due to the QR transforming from charged (Fig.1E, red region) to neutral (Fig.1E, blue region) with increasing excitation time. We confirmed this via the ratio of radiative lifetimes between the two distinct PL regions. which is confirmed by the radiative lifetime ratio between the two different PL regions (note S6). In a charged QR with electron number (n_c), the recombination lifetimes of single exciton and biexciton (including radiative and nonradiative Auger recombination) differ from those in a neutral QR due to changes in the number of nonradiative Auger pathways for single exciton and biexciton. Therefore, by constructing a charging-associated Auger recombination model (note S7), we can calculate the evolution of the n_c and nonradiative Auger lifetime of the biexciton.

In summary, while similar PL enhancement effects have been reported, our study presents a unique mechanism involving photoinduced Br ion migration and discharge

in $CsPbBr_3$ QRs. This distinguishes our findings from those of other studies focusing on ligand and defect rearrangement or photochemical reactions.

Table S1. Corresponding parameters of the QRs and QRs-cavity system:

QR Size	$14\times14\times32$ nm^3	
Single exciton	$k_\mathrm{X} = 8.77\times10^7$ s^{-1}	$\tau_\mathrm{X} = 11.37$ ns
Biexciton	$k_\mathrm{XX} = 4.24\times10^8$ s^{-1}	$\tau_\mathrm{XX} = 2.36$ ns
Biexciton<br>QR concentrations	$k_{r,\mathrm{XX}} = 3.52\times10^8$ s^{-1}	$\tau_{r,\mathrm{XX}} = 2.84$ ns
	$k_{\mathrm{A,XX}} = 7.17\times10^7$ s^{-1}	$\tau_{\mathrm{A,XX}} = 13.95$ ns
	$n_\mathrm{QD} = 0.9$ $\mu\mathrm{M}\times N_\mathrm{A}$	
Refractive index of QR solutions	$n_{ref} = 1.43$	
Lifetime of cavity photons	$\tau_c = \dfrac{2n_{ref}L}{c\cdot(-\ln(R_1R_2))} = 0.19ns$	
Saturated gain	$G_0 = 100$	
The probability of a transition per spin allowed	$\gamma = \dfrac{cG_0}{2n_{ref}n_\mathrm{QD}}$	

Table S2. Comparison of the amount of Mn (atomic %, with respect to Pb) taken as a precursor for post-synthesis doping with that obtained in the product QRs measured using inductively coupled plasma mass spectrometry (ICP-MS).

Mn-doped $CsPbBr_3$ QRs	
%Mn precursor taken	%Mn from ICP-MS
30	0.3
50	3.5
70	6.4

Table S3. Comparison of parameters of QD liquid lasers.(*7*, *8*, *19–23*, *102*, *103*) Therein, QDs, QRs and NPLs are abbreviations for quantum dots, quantum rods and nanoplatelets, respectively. Most measurements use nanosecond lasers as the pump source (gray areas), and two measurements use femtosecond pump sources (blue areas).

Year	Materials	Biexciton QY	Cavity	Qulity	Pump source	Threshold	μJ/mm^2	<*N*>	Reference
2002	QRs	\	WGM	\	ns	0.08 mJ	283	\	(*102*)
	QDs	\	WGM	\	ns	3.2 mJ	11320	\	(*102*)
2008	QDs	\	WGM	6500	ns	44 mJ/cm^2	440-530	\	(*103*)
2015	NPLs	\	WGM	1200	fs	1.2 mJ/cm^2;	12	\	(*21*)
2015	QDs	0.141-0.667	WGM	3000	ns	25.2 mJ/cm^2;	252	3.3	(*19*)
2015	QDs	\	WGM	>10^7	ns	0.1 μJ/cm^2	0.1	4.1	(*20*)
2023	QDs	\	WGM	7480	fs	22.7 μJ/cm^2	0.227	\	(*22*)
2024	QDs	\	plasmonic lattice cavity	763	fs	34 μJ/cm^2	0.34	\	(*23*)
2024	QDs	0.287	Littrow cavity	2030	ns	39 mJ/cm^2	390	>1	(*7*)
2024	QDs	0.76	Littrow cavity	1041	ns	44 mJ/cm^2	440	\	(*8*)
**Our work**	**QRs**	**0.98**	**F-P**	**1325**	**ns**	**9.86 μJ/mm^2**	**9.86**	**0.098**	
			**WGM**	**3495**	**fs**	**2.9 μJ/cm^2**	**0.029**	**0.087**	

REFERENCES

1. F. P. García De Arquer, D. V. Talapin, V. I. Klimov, Y. Arakawa, M. Bayer, E. H. Sargent, Semiconductor quantum dots: technological progress and future challenges. *Science* **373**, eaaz8541 (2021).

2. M. Liu, N. Yazdani, M. Yarema, M. Jansen, V. Wood, E. H. Sargent, Colloidal quantum dot electronics. *Nat. Electron.* **4**, 548–558 (2021).

3. A. Dey, J. Ye, A. De, E. Debroye, S. K. Ha, E. Bladt, A. S. Kshirsagar, Z. Wang, J. Yin, Y. Wang, L. N. Quan, F. Yan, M. Gao, X. Li, J. Shamsi, T. Debnath, M. Cao, M. A. Scheel, S. Kumar, J. A. Steele, M. Gerhard, L. Chouhan, K. Xu, X. Wu, Y. Li, Y. Zhang, A. Dutta, C. Han, I. Vincon, A. L. Rogach, A. Nag, A. Samanta, B. A. Korgel, C.-J. Shih, D. R. Gamelin, D. H. Son, H. Zeng, H. Zhong, H. Sun, H. V. Demir, I. G. Scheblykin, I. Mora-Seró, J. K. Stolarczyk, J. Z. Zhang, J. Feldmann, J. Hofkens, J. M. Luther, J. Pérez-Prieto, L. Li, L. Manna, M. I. Bodnarchuk, M. V. Kovalenko, M. B. J. Roeffaers, N. Pradhan, O. F. Mohammed, O. M. Bakr, P. Yang, P. Müller-Buschbaum, P. V. Kamat, Q. Bao, Q. Zhang, R. Krahne, R. E. Galian, S. D. Stranks, S. Bals, V. Biju, W. A. Tisdale, Y. Yan, R. L. Z. Hoye, L. Polavarapu, State of the art and prospects for halide perovskite nanocrystals. *ACS Nano* **15**, 10775–10981 (2021).

4. Y. S. Park, J. Roh, B. T. Diroll, R. D. Schaller, V. I. Klimov, Colloidal quantum dot lasers. *Nat. Rev. Mater.* **6**, 382–401 (2021).

5. J. Chen, W. Du, J. Shi, M. Li, Y. Wang, Q. Zhang, X. Liu, Perovskite quantum dot lasers. *InfoMat* **2**, 170–183 (2020).

6. N. Ahn, C. Livache, V. Pinchetti, H. Jung, H. Jin, D. Hahm, Y.-S. Park, V. I. Klimov, Electrically driven amplified spontaneous emission from colloidal quantum dots. *Nature* **617**, 79–85 (2023).

7. X. Lin, Y. Yang, X. Li, Y. Lv, Z. Wang, J. Du, X. Luo, D. Zhou, C. Xiao, K. Wu, Blue lasers using low-toxicity colloidal quantum dots. *Nat. Nanotechnol.* **20**, 229–236 (2025).

8. D. Hahm, V. Pinchetti, C. Livache, N. Ahn, J. Noh, X. Li, J. Du, K. Wu, V. I. Klimov, Colloidal quantum dots enable tunable liquid-state lasers. *Nat. Mater.* **24**, 48–55 (2025).

9. X. Li, Y. Jia, M. Liu, S. He, J. Guo, K. Wu, Ultrastable lasing from perovskite colloidal nanocrystals. *Sci. Adv.* **11**, eadq9002 (2025).

10. X. Li, J. Du, G. Zhao, B. Zhang, C. Livache, N. Ahn, Y. Jia, M. Li, Y. Chen, J. Zhu, J. Guo, V. I. Klimov, K. Wu, Two-color amplified spontaneous emission from Auger-suppressed quantum dots in liquids. *Adv. Mater.* **36**, e2308979 (2024).

11. D. Psaltis, S. R. Quake, C. Yang, Developing optofluidic technology through the fusion of microfluidics and optics. *Nature* **442**, 381–386 (2006).

12. Z. Li, D. Psaltis, Optofluidic dye lasers. *Microfluid. Nanofluid.* **4**, 145–158 (2008).

13. H. Schmidt, A. R. Hawkins, The photonic integration of non-solid media using optofluidics. *Nat. Photonics* **5**, 598–604 (2011).

14. M. V. Suryanarayana, Isotope separation of ^{176}Lu a precursor to ^{177}Lu medical isotope using broadband lasers. *Sci. Rep.* **11**, 6118 (2021).

15. V. I. Klimov, A. A. Mikhailovsky, S. Xu, A. Malko, J. A. Hollingsworth, C. A. Leatherdale, H.-J. Eisler, M. G. Bawendi, Optical gain and stimulated emission in nanocrystal quantum dots. *Science* **290**, 314–317 (2000).

16. O. V. Kozlov, Y.-S. Park, J. Roh, I. Fedin, T. Nakotte, V. I. Klimov, Sub–single-exciton lasing using charged quantum dots coupled to a distributed feedback cavity. *Science* **365**, 672–675 (2019).

17. F. Fan, O. Voznyy, R. P. Sabatini, K. T. Bicanic, M. M. Adachi, J. R. McBride, K. R. Reid, Y.-S. Park, X. Li, A. Jain, R. Quintero-Bermudez, M. Saravanapavanantham, M. Liu, M. Korkusinski, P. Hawrylak, V. I. Klimov, S. J. Rosenthal, S. Hoogland, E. H. Sargent, Continuous-wave lasing in colloidal quantum dot solids enabled by facet-selective epitaxy. *Nature* **544**, 75–79 (2017).

18. I. Tanghe, M. Samoli, I. Wagner, S. A. Cayan, A. H. Khan, K. Chen, J. Hodgkiss, I. Moreels, D. V. Thourhout, Z. Hens, P. Geiregat, Optical gain and lasing from bulk cadmium sulfide nanocrystals through bandgap renormalization. *Nat. Nanotechnol.* **18**, 1423–1429 (2023).

19. Y. Wang, K. S. Leck, V. D. Ta, R. Chen, V. Nalla, Y. Gao, T. He, H. V. Demir, H. Sun, Blue liquid lasers from solution of CdZnS/ZnS ternary alloy quantum dots with quasi-continuous pumping. *Adv. Mater.* **27**, 169–175 (2015).

20. A. Kiraz, Q. Chen, X. Fan, Optofluidic lasers with aqueous quantum dots. *ACS Photonics* **2**, 707–713 (2015).

21. M. Li, M. Zhi, H. Zhu, W.-Y. Wu, Q.-H. Xu, M. H. Jhon, Y. Chan, Ultralow-threshold multiphoton-pumped lasing from colloidal nanoplatelets in solution. *Nat. Commun.* **6**, 8513 (2015).

22. Y. Wu, Z. Huang, Q. Sun, V. D. Ta, S. Wang, Y. Wang, A new generation of liquid lasers from engineered semiconductor nanocrystals with giant optical gain. *Laser Photonics Rev.* **17**, 2200703 (2022).

23. M. J. H. Tan, S. K. Patel, J. Chiu, Z. T. Zheng, T. W. Odom, Liquid lasing from solutions of ligand-engineered semiconductor nanocrystals. *J. Chem. Phys.* **160**, 154703 (2024).

24. K. Wu, Y. S. Park, J. Lim, V. I. Klimov, Towards zero-threshold optical gain using charged semiconductor quantum dots. *Nat. Nanotechnol.* **12**, 1140–1147 (2017).

25. J. J. Geuchies, B. Brynjarsson, G. Grimaldi, S. Gudjonsdottir, W. van der Stam, W. H. Evers, A. J. Houtepen, Quantitative electrochemical control over optical gain in quantum-dot solids. *ACS Nano* **15**, 377–386 (2021).

26. K. Wu, J. Lim, V. I. Klimov, Superposition principle in Auger recombination of charged and neutral multicarrier states in semiconductor quantum dots. *ACS Nano* **11**, 8437–8447 (2017).

27. Q. Li, Y. Yang, W. Que, T. Lian, Size- and morphology-dependent Auger recombination in $CsPbBr_3$ perovskite two-dimensional nanoplatelets and one-dimensional nanorods. *Nano Lett.* **19**, 5620–5627 (2019).

28. C. Zhang, J. Chen, S. Wang, L. Kong, S. W. Lewis, X. Yang, A. L. Rogach, G. Jia, Metal halide perovskite nanorods: Shape matters. *Adv. Mater.* **32**, 2002736 (2020).

29. H. Htoon, J. A. Hollingsworth, R. Dickerson, V. I. Klimov, Effect of zero- to one-dimensional transformation on multiparticle Auger recombination in semiconductor quantum rods. *Phys. Rev. Lett.* **91**, 227401 (2003).

30. X. Han, Z. Huang, G. Zhang, C. Yang, J. Li, M. Zhang, Z. Chen, J. Wang, R. Chen, C. Qin, J. Hu, Z. Yang, X. Liu, Y. Wang, J. Ma, L. Xiao, S. Jia, Ultrafast exciton formation in perovskite quantum rods. *Nano Lett.* **25**, 4913–4920 (2025).

31. A. Sitt, A. Salant, G. Menagen, U. Banin, Highly emissive nano rod-in-rod heterostructures with strong linear polarization. *Nano Lett.* **11**, 2054–2060 (2011).

32. X. Bai, H. Li, Y. Peng, G. Zhang, C. Yang, W. Guo, X. Han, J. Li, R. Chen, C. Qin, J. Hu, G. Yang, H. Zhong, L. Xiao, S. Jia, Role of aspect ratio in the photoluminescence of single CdSe/CdS dot-in-rods. *J. Phys. Chem. C* **126**, 2699–2707 (2022).

33. J. Ye, N. Mondal, B. P. Carwithen, Y. Zhang, L. Dai, X.-B. Fan, J. Mao, Z. Cui, P. Ghosh, C. Otero-Martínez, L. van Turnhout, Y.-T. Huang, Z. Yu, Z. Chen, N. C. Greenham, S. D. Stranks, L. Polavarapu, A. Bakulin, A. Rao, R. L. Z. Hoye, Extending the defect tolerance of halide perovskite nanocrystals to hot carrier cooling dynamics. *Nat. Commun.* **15**, 8120 (2024).

34. S. ten Brinck, F. Zaccaria, I. Infante, Defects in lead halide perovskite nanocrystals: Analogies and (many) differences with the bulk. *ACS Energy Lett.* **4**, 2739–2747 (2019).

35. C. Yang, G. Zhang, Y. Gao, B. Li, X. Han, J. Li, M. Zhang, Z. Chen, Y. Wei, R. Chen, C. Qin, J. Hu, Z. Yang, G. Zeng, L. Xiao, S. Jia, Size-dependent photoluminescence blinking mechanisms and volume scaling of biexciton Auger recombination in single $CsPbI_3$ perovskite quantum dots. *J. Chem. Phys.* **160**, 174505 (2024).

36. C. Yang, Y. Li, X. Hou, M. Zhang, G. Zhang, B. Li, W. Guo, X. Han, X. Bai, J. Li, R. Chen, C. Qin, J. Hu, L. Xiao, S. Jia, Conversion of photoluminescence blinking types in single colloidal quantum dots. *Small* **20**, e2309134 (2024).

37. B. Li, G. Zhang, Y. Zhang, C. Yang, W. Guo, Y. Peng, R. Chen, C. Qin, Y. Gao, J. Hu, R. Wu, J. Ma, H. Zhong, Y. Zheng, L. Xiao, S. Jia, Biexciton dynamics in single colloidal CdSe quantum dots. *J. Phys. Chem. Lett.* **11**, 10425–10432 (2020).

38. G. Yuan, C. Ritchie, M. Ritter, S. Murphy, D. E. Gómez, P. Mulvaney, The degradation and blinking of single $CsPbI_3$ perovskite quantum dots. *J. Phys. Chem. C* **122**, 13407–13415 (2018).

39. X. Han, G. Zhang, B. Li, C. Yang, W. Guo, X. Bai, P. Huang, R. Chen, C. Qin, J. Hu, Y. Ma, H. Zhong, L. Xiao, S. Jia, Blinking mechanisms and intrinsic quantum-confined Stark effect in single methylammonium lead bromide perovskite quantum dots. *Small* **16**, e2005435 (2020).

40. X. Chen, Z. Sun, B. Cai, X. Li, S. Zhang, D. Fu, Y. Zou, Z. Fan, H. Zeng, Substantial improvement of operating stability by strengthening metal-halogen bonds in halide perovskites. *Adv. Funct. Mater.* **32**, 2112129 (2022).

41. S. Feldmann, M. K. Gangishetty, I. Bravić, T. Neumann, B. Peng, T. Winkler, R. H. Friend, B. Monserrat, D. N. Congreve, F. Deschler, Charge carrier localization in doped perovskite nanocrystals enhances radiative recombination. *J. Am. Chem. Soc.* **143**, 8647–8653 (2021).

42. J. Park, Y. H. Won, T. Kim, E. Jang, D. Kim, Electrochemical charging effect on the optical properties of InP/ZnSe/ZnS quantum dots. *Small* **16**, 2003542 (2020).

43. B. Li, G. Zhang, Y. Gao, X. Chen, R. Chen, C. Qin, J. Hu, R. Wu, L. Xiao, S. Jia, Single quantum dot spectroscopy for exciton dynamics. *Nano Res.* **17**, 10332–10345 (2024).

44. C. Yang, G. Zhang, J. Li, R. Chen, C. Qin, J. Hu, Z. Yang, L. Xiao, S. Jia, Mechanisms and suppression of quantum dot blinking. *Laser Photonics Rev.* **19**, 2402269 (2025).

45. J. Li, D. Wang, G. Zhang, C. Yang, W. Guo, X. Han, X. Bai, R. Chen, C. Qin, J. Hu, L. Xiao, S. Jia, The role of surface charges in the blinking mechanisms and quantum-confined Stark effect of single colloidal quantum dots. *Nano Res.* **15**, 7655–7661 (2022).

46. Y. Yang, J. Li, Y. Li, B. Li, J. Zhang, Z. Chen, L. Xiao, R. Lai, Z. Zeng, G. Zhang, B. Ji, High-purity single-photon emission in near-infrared InAs colloidal quantum dots with strong exciton confinement. *Nano Lett.* **25**, 7595–7602 (2025).

47. B. Li, H. Huang, G. Zhang, C. Yang, W. Guo, R. Chen, C. Qin, Y. Gao, V. P. Biju, A. L. Rogach, L. Xiao, S. Jia, Excitons and biexciton dynamics in single $CsPbBr_3$ perovskite quantum dots. *J. Phys. Chem. Lett.* **9**, 6934–6940 (2018).

48. W. Guo, J. Tang, G. Zhang, B. Li, C. Yang, R. Chen, C. Qin, J. Hu, H. Zhong, L. Xiao, S. Jia, Photoluminescence blinking and biexciton Auger recombination in single colloidal quantum dots with sharp and smooth core/shell interfaces. *J. Phys. Chem. Lett.* **12**, 405–412 (2021).

49. J. Zhu, X. Yang, Y. Zhu, Y. Wang, J. Cai, J. Shen, L. Sun, C. Li, Room-temperature synthesis of Mn-doped cesium lead halide quantum dots with high Mn substitution ratio. *J. Phys. Chem. Lett.* **8**, 4167–4171 (2017).

50. Z. Huang, Q. Sun, S. Wang, H. Shen, W. Cai, Y. Wang, Broadband tunable optical gain from ecofriendly semiconductor quantum dots with near-half-exciton threshold. *Nano Lett.* **23**, 4032–4038 (2023).

51. J. Sun, Z. Zhang, Y. Chen, M. Qiu, W. Jin, C.-Z. Ning, H. J. Snaith, A. K.-Y. Jen, D. Lei, Quantum-defect-minimized, three-photon-pumped ultralow-threshold perovskite excitonic lasing. *Adv. Funct. Mater.* **34**, 2401247 (2024).

52. Z. Huang, Y. Ren, Y. Wu, J. Qin, K. Jiao, H. Zhang, V. D. Ta, F. Gao, H. Shen, Y. Wang, Sustained continuous-wave lasing in quantum dot microfluids. *Adv. Mater.* **37**, e09457 (2025).

53. Y. Wu, H. Zhu, Z. Huang, H. Zhang, Y. Ren, H. Shen, Y. Wu, Y. Wang, Unlocking megawatt-peak-power laser emission with colloidal quantum dots. *Sci. Adv.* **11**, eaea8326 (2025).

54. T.-L. Shen, H.-W. Hu, W.-J. Lin, Y.-M. Liao, T.-P. Chen, Y.-K. Liao, T.-Y. Lin, Y.-F. Chen, Coherent Förster resonance energy transfer: A new paradigm for electrically driven quantum dot random lasers. *Sci. Adv.* **6**, eaba1705 (2020).

55. K. P. Bera, C. Hanmandlu, H.-I. Lin, R. Ghosh, V. K. Gudelli, C.-S. Lai, C.-W. Chu, Y.-F. Chen, Fabry–Perot oscillation and resonance energy transfer: mechanism for ultralow-threshold optically and electrically driven random laser in quasi-2D ruddlesden–popper perovskites. *ACS Nano* **17**, 5373–5386 (2023).

56. S. Li, D. Lei, W. Ren, X. Guo, S. Wu, Y. Zhu, A. L. Rogach, M. Chhowalla, A. K.-Y. Jen, Water-resistant perovskite nanodots enable robust two-photon lasing in aqueous environment. *Nat. Commun.* **11**, 1192 (2020).

57. L. He, Ş. K. Özdemir, L. Yang, Whispering gallery microcavity lasers. *Laser Photonics Rev.* **7**, 60–82 (2013).

58. Y. S. Park, W. K. Bae, T. Baker, J. Lim, V. I. Klimov, Effect of Auger recombination on lasing in heterostructured quantum dots with engineered core/shell interfaces. *Nano Lett.* **15**, 7319–7328 (2015).

59. O. Svelto, *Principles of Lasers* (Springer US, 2010).

60. P. Huang, S. Sun, H. Lei, Y. Zhang, H. Qin, H. Zhong, Nonlocal interaction enhanced biexciton emission in large $CsPbBr_3$ nanocrystals. *eLight* **3**, 10 (2023).

61. G. Mannino, I. Deretzis, E. Smecca, A. La Magna, A. Alberti, D. Ceratti, D. Cahen, Temperature-dependent optical band gap in $CsPbBr_3$, $MAPbBr_3$, and $FAPbBr_3$ single crystals. *J. Phys. Chem. Lett.* **11**, 2490–2496 (2020).

62. X. Zhang, J.-X. Shen, C. G. Van de Walle, First-principles simulation of carrier recombination mechanisms in halide perovskites. *Adv. Energy Mater.* **10**, 1902830 (2020).

63. M. Zhang, X. Han, C. Yang, G. Zhang, W. Guo, J. Li, Z. Chen, B. Li, R. Chen, C. Qin, J. Hu, Z. Yang, G. Zeng, L. Xiao, S. Jia, Size Uniformity of $CsPbBr_3$ Perovskite Quantum Dots Via Manganese-Doping. *Nanomaterials* **14**, 1284 (2024).

64. B. Li, H. Huang, G. Zhang, C. Yang, W. Guo, R. Chen, C. Qin, Y. Gao, V. P. Biju, A. L. Rogach, L. Xiao, S. Jia, Excitons and biexciton dynamics in single $CsPbBr_3$ perovskite quantum dots. *J. Phys. Chem. Lett.* **9**, 6934–6940 (2018).

65. W. Guo, J. Li, B. Li, W. Zhang, C. Yang, X. Han, Z. Chen, G. Yang, Z. Yang, R. Chen, C. Qin, J. Hu, G. Zhang, L. Xiao, Measurement of absolute biexciton quantum yields in single quantum dots: breaking the limitation of weak excitation conditions. *Appl. Phys. Lett.* **127**, 052104 (2025).

66. B. Li, G. Zhang, Y. Zhang, C. Yang, W. Guo, Y. Peng, R. Chen, C. Qin, Y. Gao, J. Hu, R. Wu, J. Ma, H. Zhong, Y. Zheng, L. Xiao, S. Jia, Biexciton dynamics in single colloidal CdSe quantum dots. *J. Phys. Chem. Lett.* **11**, 10425–10432 (2020).

67. P. Spinicelli, S. Buil, X. Quélin, B. Mahler, B. Dubertret, J.-P. Hermier, Bright and grey states in CdSe-CdS nanocrystals exhibiting strongly reduced blinking. *Phys. Rev. Lett.* **102**, 136801 (2009).

68. Q. Li, Y. Yang, W. Que, T. Lian, Size- and morphology-dependent Auger recombination in $CsPbBr_3$ perovskite two-dimensional nanoplatelets and one-dimensional nanorods. *Nano Lett.* **19**, 5620–5627 (2019).

69. H. Htoon, J. A. Hollingsworth, R. Dickerson, V. I. Klimov, Effect of zero- to one-dimensional transformation on multiparticle Auger recombination in semiconductor quantum rods. *Phys. Rev. Lett.* **91**, 227401 (2003).

70. B. Li, G. Zhang, C. Yang, Z. Li, R. Chen, C. Qin, Y. Gao, H. Huang, L. Xiao, S. Jia, Fast recognition of single quantum dots from high multi-exciton emission and clustering effects. *Opt. Express* **26**, 4674–4685 (2018).

71. J. M. Pietryga, Y. S. Park, J. Lim, A. F. Fidler, W. K. Bae, S. Brovelli, V. I. Klimov, Spectroscopic and device aspects of nanocrystal quantum dots. *Chem. Rev.* **116**, 10513–10622 (2016).

72. H. Jung, N. Ahn, V. I. Klimov, Prospects and challenges of colloidal quantum dot laser diodes. *Nat. Photonics* **15**, 643–655 (2021).

73. T. Gao, Z. Wei, S. Gao, J. Shi, J. Sun, Defect-assisted Auger recombination in graphitic carbon nitride revealed by excitation-density-dependent transient absorption spectroscopy. *Opt. Lett.* **50**, 3792–3795 (2025).

74. J. Sun, D. Zhu, J. Zhao, M. Ikezawa, X. Wang, Y. Masumoto, Ultrafast carrier dynamics in $CuInS_2$ quantum dots. *Appl. Phys. Lett.* **104**, 023118 (2014).

75. V. I. Klimov, A. A. Mikhailovsky, S. Xu, A. Malko, J. A. Hollingsworth, C. A. Leatherdale, H.-J. Eisler, M. G. Bawendi, Optical gain and stimulated emission in nanocrystal quantum dots. *Science* **290**, 314–317 (2000).

76. Y.-S. Park, J. Roh, B. T. Diroll, R. D. Schaller, V. I. Klimov, Colloidal quantum dot lasers. *Nat. Rev. Mater.* **6**, 382–401 (2021).

77. B. Li, G. Zhang, C. Yang, Z. Li, R. Chen, C. Qin, Y. Gao, H. Huang, L. Xiao, S. Jia, Fast recognition of single quantum dots from high multi-exciton emission and clustering effects. *Opt. Express* **26**, 4674–4685 (2018).

78. X. Chen, Z. Sun, B. Cai, X. Li, S. Zhang, D. Fu, Y. Zou, Z. Fan, H. Zeng, Substantial improvement of operating stability by strengthening metal-halogen bonds in halide perovskites. *Adv. Funct. Mater.* **32**, 2112129 (2022).

79. O. V. Kozlov, Y.-S. Park, J. Roh, I. Fedin, T. Nakotte, V. I. Klimov, Sub–Single-exciton lasing using charged quantum dots coupled to a distributed feedback cavity. *Science* **365**, 672–675 (2019).

80. K. Wu, Y.-S. Park, J. Lim, V. I. Klimov, Towards zero-threshold optical gain using charged semiconductor quantum dots. *Nat. Nanotechnol.* **12**, 1140–1147 (2017).

81. I. du Fossé, S. Lal, A. N. Hossaini, I. Infante, A. J. Houtepen, Effect of ligands and solvents on the stability of electron charged CdSe colloidal quantum dots. *J. Phys. Chem. C* **125**, 23968–23975 (2021).

82. N. Ahn, C. Livache, V. Pinchetti, V. I. Klimov, Colloidal semiconductor nanocrystal lasers and laser diodes. *Chem. Rev.* **123**, 8251–8296 (2023).

83. C.-H. Lu, G. V. Biesold-McGee, Y. Liu, Z. Kang, Z. Lin, Doping and ion substitution in colloidal metal halide perovskite nanocrystals. *Chem. Soc. Rev.* **49**, 4953–5007 (2020).

84. W. Liu, G. Yuan, Y. Zhang, Q. Wang, S. Zhao, Z. Liu, T. Wei, J. Wang, J. Li, Localized exciton emission in $CsPbBr_3$ nanocrystals synthesized with excess bromide ions. *J. Mater. Chem. C* **7**, 10783–10788 (2019).

85. S. Tan, T. Huang, Y. Yang, Defect passivation of perovskites in high efficiency solar cells. *J. Phys. Energy* **3**, 042003 (2021).

86. S. G. Motti, D. Meggiolaro, S. Martani, R. Sorrentino, A. J. Barker, F. De Angelis, A. Petrozza, Defect activity in lead halide perovskites. *Adv. Mater.* **31**, 1901183 (2019).

87. J. Ye, M. M. Byranvand, C. O. Martínez, R. L. Z. Hoye, M. Saliba, L. Polavarapu, Defect passivation in lead-halide perovskite nanocrystals and thin films: toward efficient LEDs and solar cells. *Angew. Chem. Int. Ed. Engl.* **133**, 21804–21828 (2021).

88. J. Kang, L.-W. Wang, High defect tolerance in lead halide perovskite $CsPbBr_3$. *J. Phys. Chem. Lett.* **8**, 489–493 (2017).

89. H. Asami, Y. Abe, T. Ohtsu, I. Kamiya, M. Hara, Surface state analysis of photobrightening in CdSe nanocrystal thin films. *J. Phys. Chem. B* **107**, 12566–12568 (2003).

90. L. J. Ruan, B. Tang, A. Shu, C. Qin, Y. Ma, Self-passivation of $CsPbBr_3$ nanocrystals through introducing bromide vacancies and ultraviolet irradiation. *J. Phys. Chem. C* **125**, 1010–1017 (2021).

91. S. R. Cordero, P. J. Carson, R. A. Estabrook, G. F. Strouse, S. K. Buratto, Photo-activated luminescence of CdSe quantum dot monolayers. *J. Phys. Chem. B* **104**, 12137–12142 (2000).

92. A. Y. Nazzal, X. Wang, L. Qu, W. Yu, Y. Wang, X. Peng, M. Xiao, Environmental effects on photoluminescence of highly luminescent CdSe and CdSe/ZnS core/shell nanocrystals in polymer thin films. *J. Phys. Chem. B* **108**, 5507–5515 (2004).

93. K. Pechstedt, T. Whittle, J. Baumberg, T. Melvin, Photoluminescence of Colloidal CdSe/ZnS quantum dots: The critical effect of water molecules. *J. Phys. Chem. C* **114**, 12069–12077 (2010).

94. D. Hong, Y. Zhang, S. Pan, H. Liu, W. Mao, Z. Lu, Y. Tian, Moisture-dependent blinking of individual $CsPbBr_3$ nanocrystals revealed by single-particle spectroscopy. *J. Phys. Chem. Lett.* **13**, 10751–10758 (2022).

95. S. Dembski, C. Graf, T. Krüger, U. Gbureck, A. Ewald, A. Bock, E. Rühl, Photoactivation of CdSe/ZnS quantum dots embedded in silica colloids. *Small* **4**, 1516–1526 (2008).

96. T. Zhang, H. Zhao, D. Riabinina, M. Chaker, D. Ma, Concentration-dependent photoinduced photoluminescence enhancement in colloidal PbS quantum dot solution. *J. Phys. Chem. C* **114**, 10153–10159 (2010).

97. Z. Hu, S. Liu, H. Qin, J. Zhou, X. Peng, Oxygen stabilizes photoluminescence of CdSe/CdS core/shell quantum dots via deionization. *J. Am. Chem. Soc.* **142**, 4254–4264 (2020).

98. R. Kusterer, S. Krohn, M. Wehrmeister, C. Strelow, T. Kipp, A. Mews, A closer look at the effects of oxygen on the photoluminescence properties of CdSe/CdS quantum dots. *J. Chem. Phys.* **161**, 024706 (2024).

99. V. Biju, R. Kanemoto, Y. Matsumoto, S. Ishii, S. Nakanishi, T. Itoh, Y. Baba, M. Ishikawa, Photoinduced photoluminescence variations of CdSe quantum dots in polymer solutions. *J. Phys. Chem. C* **111**, 7924–7932 (2007).

100. S. Impellizzeri, B. McCaughan, J. F. Callan, F. M. Raymo, Photoinduced enhancement in the luminescence of hydrophilic quantum dots coated with photocleavable ligands. *J. Am. Chem. Soc.* **134**, 2276–2283 (2012).

101. V. Dzhagan, O. Stroyuk, O. Raievska, O. Isaieva, O. Kapush, O. Selyshchev, V. Yukhymchuk, M. Valakh, D. R. T. Zahn, Photoinduced enhancement of photoluminescence of colloidal II-VI nanocrystals in polymer matrices. *Nanomaterials* **10**, 2565 (2020).

102. M. Kazes, D. Y. Lewis, Y. Ebenstein, T. Mokari, U. Banin, Lasing from semiconductor quantum rods in a cylindrical microcavity. *Adv. Mater.* **14**, 317–321 (2002).

103. J. Schäfer, J. P. Mondia, R. Sharma, Z. H. Lu, A. S. Susha, A. L. Rogach, L. J. Wang, Quantum dot microdrop laser. *Nano Lett.* **8**, 1709–1712 (2008).
